# Accumulation of Squalene in a Microalga *Chlamydomonas reinhardtii* by Genetic Modification of Squalene Synthase and Squalene Epoxidase Genes

**DOI:** 10.1371/journal.pone.0120446

**Published:** 2015-03-12

**Authors:** Masataka Kajikawa, Seiko Kinohira, Akira Ando, Miki Shimoyama, Misako Kato, Hideya Fukuzawa

**Affiliations:** 1 Graduate School of Biostudies, Kyoto University, Kyoto, Japan; 2 Graduate School of Humanities and Science, Ochanomizu University, Tokyo, Japan; Nanyang Technological University, SINGAPORE

## Abstract

Several microalgae accumulate high levels of squalene, and as such provide a potentially valuable source of this useful compound. However, the molecular mechanism of squalene biosynthesis in microalgae is still largely unknown. We obtained the sequences of two enzymes involved in squalene synthesis and metabolism, squalene synthase (CrSQS) and squalene epoxidase (CrSQE), from the model green alga *Chlamydomonas reinhardtii*. CrSQS was functionally characterized by expression in *Escherichia coli* and CrSQE by complementation of a budding yeast *erg1* mutant. Transient expression of CrSQS and CrSQE fused with fluorescent proteins in onion epidermal tissue suggested that both proteins were co-localized in the endoplasmic reticulum. *CrSQS*-overexpression increased the rate of conversion of ^14^C-labeled farnesylpyrophosphate into squalene but did not lead to over-accumulation of squalene. Addition of terbinafine caused the accumulation of squalene and suppression of cell survival. On the other hand, in *CrSQE*-knockdown lines, the expression level of *CrSQE* was reduced by 59–76% of that in wild-type cells, and significant levels of squalene (0.9–1.1 μg mg^–1^ cell dry weight) accumulated without any growth inhibition. In co-transformation lines with *CrSQS*-overexpression and *CrSQE*-knockdown, the level of squalene was not increased significantly compared with that in solitary *CrSQE*-knockdown lines. These results indicated that partial knockdown of *CrSQE* is an effective strategy to increase squalene production in *C*. *reinhardtii* cells.

## Introduction

Squalene is an important intermediate of sterol biosynthesis in a variety of organisms from bacteria to humans [1]. Squalene has been used as a natural antioxidant, adjuvant for vaccines, dietary supplement and skin moisturizer for therapeutic, pharmacological and cosmetic purposes [1,2]. The main source of squalene is shark liver oil, and alternative sources are desired in order to secure a sustainable supply of squalene and for the conservation of marine organisms [1]. In microorganisms, squalene content shows wide variety depending on the species. For instance, squalene contents from *Saccharomyces cerevisiae* and *Torulaspora delbrueckii* were found to be 0.04 and 0.24 μg mg^−1^ respectively, dry weight of cells [3]. In microalgae *Chlamydomonas reinhardtii* (in this study) and *Phaeodactylum tricornutum* [4], squalene was not detected in the wild-type cells. In contrast, *Aurantiochytrium mangrovei* accumulates squalene (0.2–0.4 μg mg^−1^ dry weight) [5,6]. The *Botryococcus braunii* and *Euglena* are also reported to accumulate squalene in the cells [1]. Recently, *Aurantiochytrium* sp. strain 18W-13a was reported to accumulate huge amount of squalene (171 μg mg^−1^ dry weight) in cells [7] and is expected to be useable as an industrial source of this useful compound. For further accumulation of squalene or controls of squalene production in microalgal cells, genetic modification of genes involved in squalene metabolism are required. However, transformation system is not available in the most microalgae including *Aurantiochytrium* sp. and only a few examples of the characterization of genes directly responsible for squalene metabolism have been reported in microalgae [8,9].

In land plants, squalene is produced via two pathways, the mevalonate (MVA) pathway and the methylerythritol phosphate (MEP) pathway [1]. In contrast, green algae have only the MEP pathway [1,10,11]. In the model green microalga *C*. *reinhardtii*, squalene is predicted to be synthesized via the MEP pathway, as in other green algae [12]. However, no enzymes in the MEP pathway of *C*. *reinhardtii* have been characterized as yet. In *C*. *reinhardtii*, two major sterols, ergosterol and 7-dehydroporiferasterol, are produced from squalene via two branched pathways [13–16]. In the first step of the pathway, squalene is produced from farnesyl diphosphate (FPP) by squalene synthase (SQS) and converted to 2,3-oxidosqualene by squalene epoxidase (SQE).

SQS catalyzes the reductive dimerization of two molecules of FPP in a two-step reaction to produce squalene. This reaction proceeds via head-to-head coupling of two FPP molecules to form squalene via a stable cyclopropylcarbinyl diphosphate intermediate [17]. SQS genes have been characterized from many organisms, such as yeast, rat, human and land plants [1]. The enzymatic activities of SQS in two oil-producing microalgae, *Botryococcus braunii* [8] and *Aurantiochytrium* sp. KRS101 [9], have been characterized *in vitro*. However, there have been no reports about the *in vivo* functional analysis of SQS in algae. On the other hand, SQE catalyzes the insertion of an oxygen atom across a C-C double bond to form an epoxide in a P450-type reaction. SQE genes have also been characterized from many organisms, such as yeast, rat, human and land plants [1], but not yet from algae.

In order to understand the functional importance of genes encoding enzymes directly responsible for squalene synthesis and metabolism in squalene accumulation in microalgal cells, we describe here the isolation and characterization of putative *C*. *reinhardtii* SQS and SQE genes. Transgenic studies using these genes in *C*. *reinhardtii* cells revealed that partial knockdown (KD) of *CrSQE* expression enhanced the accumulation of significant amounts of squalene without any growth inhibition. In contrast, overexpression of *CrSQS* did not influence the amount of squalene in cells.

## Results

### Identification of *SQS* and *SQE* genes in *C*. *reinhardtii*

Two genes, *Cre03*.*g175250* and *Cre17*.*g734644*, encoding putative enzymes involved in sterol metabolism were predicted in the *C*. *reinhardtii* genome database v5.5 (Phytozome v10; http://phytozome.jgi.doe.gov/pz/portal.html#!info?alias=Org_Creinhardtii) [18,19]. The predicted amino acid sequences showed high levels of similarity with SQS and SQE enzymes in other organisms, respectively. Therefore, these genes were prospectively designated as *CrSQS* and *CrSQE*, respectively. CrSQS shared 50% sequence identity with *Arabidopsis thaliana* SQS1 (AtSQS1) [20–22], 47% with *B*. *braunii* SQS (BbSQS) [8], 41% with human SQS (HsSQS) [17,23–25], and 34% with *S*. *cerevisiae* SQS (ScERG9) [23] (S1A Fig.). CrSQE shared 51% sequence identity with the *A*. *thaliana* SQE1 (AtSQE1) [26], 36% with human SQE (HsSQE) [27], 34% with rat SQE (RnSQE) [28] and 33% with *S*. *cerevisiae* SQE (ScERG1) [29,30] (S1B Fig.). Southern blotting analysis indicated that these genes are present as single-copy genes in *C*. *reinhardtii* (S2 Fig.). The SOSUI program (http://harrier.nagahama-i-bio.ac.jp/sosui/sosui_submit.html) [31] predicted that the CrSQS protein has two putative transmembrane domains (TMDs) at the C-terminus (Phe-398 to Glu-420 and Gln-436 to Leu-458) (S1A Fig.). Likewise, one TMD at the N-terminus (Pro-29 to Ala-51) and two TMDs at the C-terminus (Ala-424 to Phe-446 and Leu-472 to Phe-494) were predicted in the CrSQE protein (S1B Fig.). The above results suggested that the *CrSQS* and *CrSQE* genes could encode membrane-bound SQS and SQE, respectively, involved in the squalene synthesis and sterol metabolism in *C*. *reinhardtii*.

### *In vitro* enzymatic activity of recombinant CrSQS protein

**Fig 1:**
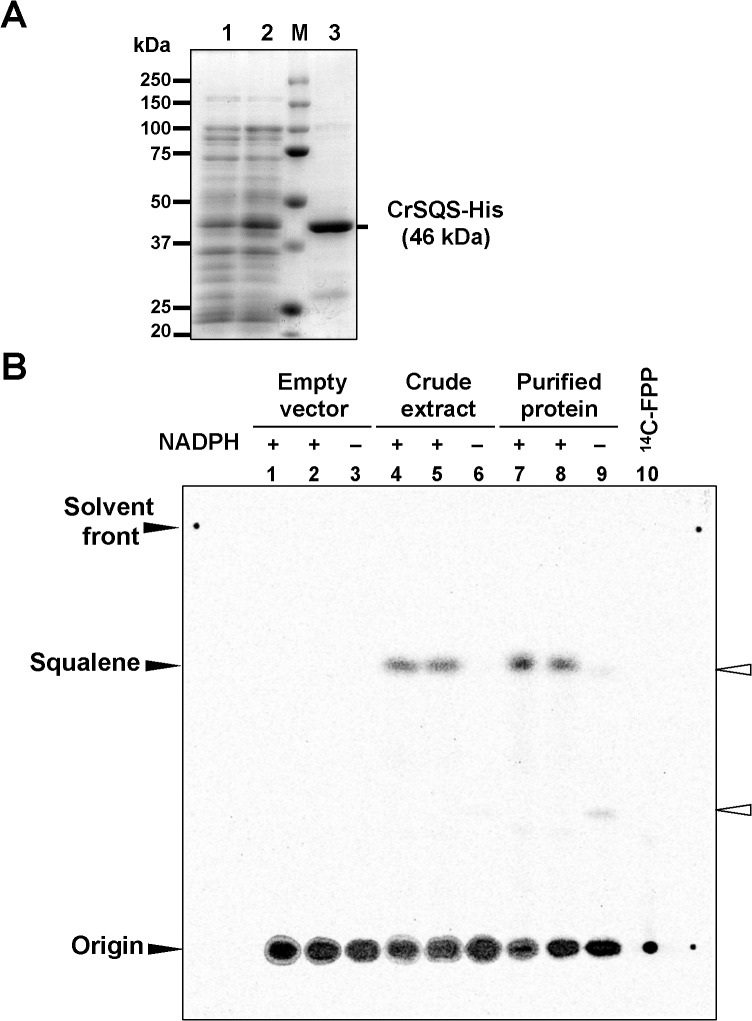
Functional characterization of CrSQS in *E*. *coli*. A) SDS-polyacrylamide gel electrophoresis of crude extracts from cells harboring the empty vector pET-21b (lane 1), cells expressing *CrSQS* (lane 2) and purified CrSQS using a TALON Metal affinity chromatography. B) Normal-phase thin-layer chromatogram of the reaction products derived from (1–^14^C) farnesyl diphosphate (FPP). Crude extracts from cells harboring the empty vector (lanes 1–3), from cells expressing CrSQS (lanes 4–6) and purified CrSQS protein (lanes 7–9) were assayed for SQS activity using (1–^14^C) FPP and Mg^2+^ with NADPH (lanes 1–2, 4–5, and 7–8 are experimental replicates) or without NADPH (lanes 3, 6, 9). Authentic (1–^14^C) FPP was loaded in lane 10 as a negative control. The positions of origin, solvent front and authentic squalene are indicated on the left. Open triangles on the right indicate position of signals from putative dehydrosqualene and 12-hydroxysqualene in lane 9.

To confirm that *CrSQS* encodes a functional SQS, CrSQS was expressed as a fusion protein with a His-tag at the C-terminus in *Escherichia coli*. The sequence encoding the C-terminal 64 amino acid residues, which included the hydrophobic transmembrane regions, was removed, and the remaining 1,191-bp fragment encoding 397 amino acid residues (Met-1 to Asp-397) cloned upstream of the 6×His-tag sequence in a pET-21b vector. A 46-kDa band, which corresponded to the predicted size of the recombinant CrSQS protein, was detected in the soluble protein fraction of IPTG-induced *E*. *coli* cells harboring the *CrSQS*-expression plasmid, but not in extracts from cells harboring the empty vector (Fig. 1A). The recombinant CrSQS protein was purified from the soluble protein fractions by Co^2+^-affinity (TALON) column chromatography (lane 3 in Fig. 1A).

To evaluate squalene synthase activity of CrSQS, the crude soluble extract and purified CrSQS protein were used for enzyme assays in the presence of (1–^14^C) FPP, nicotinamide adenine dinucleotide phosphate (NADPH) and Mg^2+^ as a cofactor. Normal-phase thin layer chromatography (TLC) analysis was performed to separate the reaction products. ^14^C-labeled squalene was detected in samples containing the crude extract or purified CrSQS protein (lanes 4, 5 and 7, 8 in Fig. 1B) but not in a sample from cells containing the empty vector (lanes 1–3 in Fig. 1B). These results indicated that CrSQS protein had SQS activity. When NADPH was removed, radiolabeled squalene was not detected in any sample (lane 3, 6 and 9 in Fig. 1B). Instead, two weak signals, which might be dehydrosqualene and 12-hydroxysqualene [20,22], were detected when purified CrSQS was incubated with (1–^14^C) FPP but without NADPH (lane 9 in Fig. 1B). To further characterize the enzymatic activity, crude and purified CrSQS protein was incubated in a mixture containing (1–^14^C) FPP, with or without NADPH, and either Mg^2+^ or Mn^2+^ as a cofactor, and the reaction products were separated by normal and reverse-phase TLC (S3 Fig.). TLC analyses indicated that CrSQS protein showed SQS activity in the presence of NADPH and either Mg^2+^ or Mn^2+^ (lanes 1 to 4 in S3 Fig.).

### Complementation analysis of *CrSQE* in a yeast *erg1* mutant

**Fig 2:**
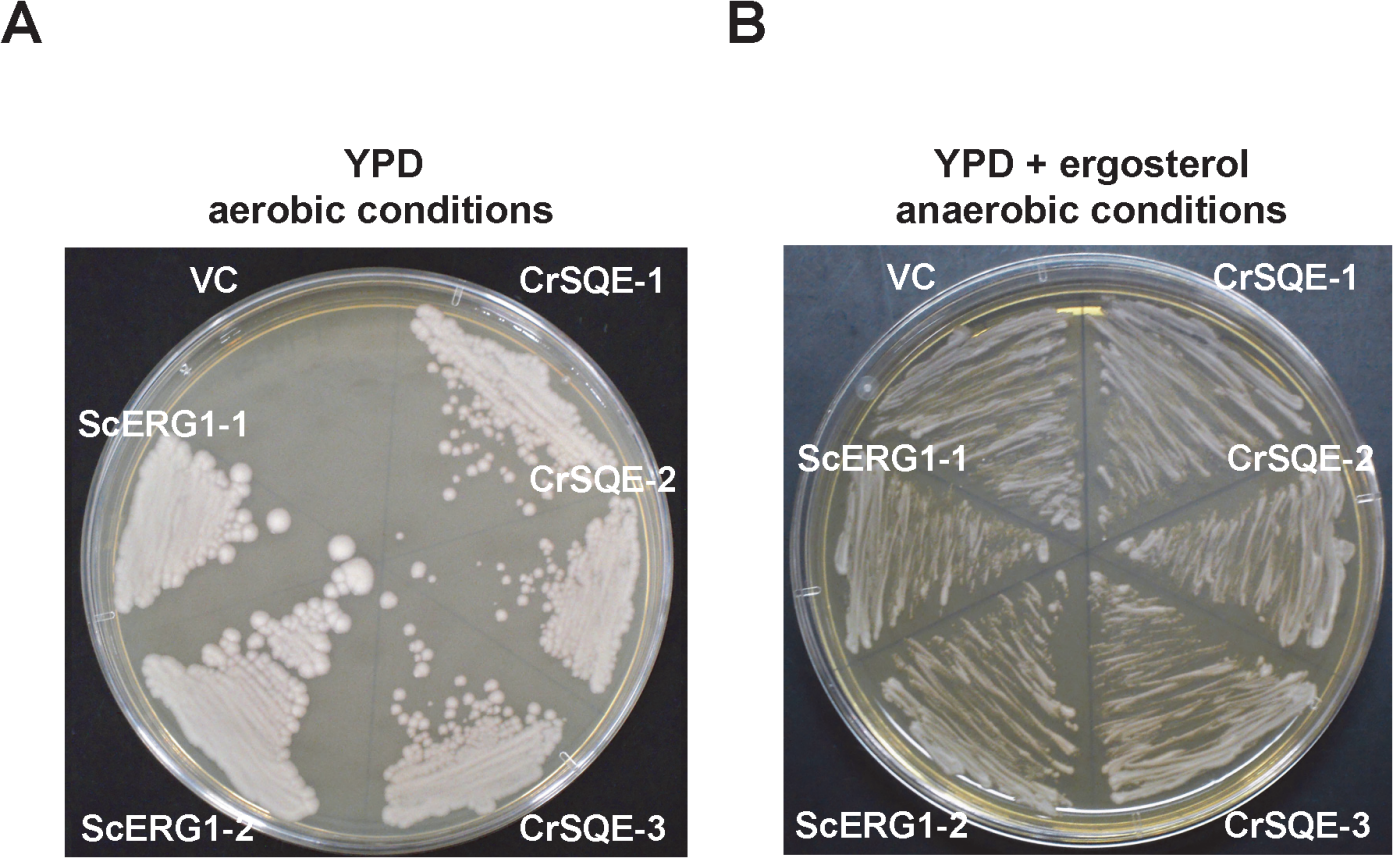
Complementation of a yeast *erg1* mutant KLN1 by *CrSQE* expression. Three independent lines harboring the pAUR123-CrSQE (CrSQE-1 to CrSQE-3), two yeast lines harboring the pAUR123-ScERG1 (ScERG1-1 and ScERG1-2), and a control line harboring an empty pAUR123 vector (VC) were streaked onto YPD medium without ergosterol under the aerobic conditions (A) or with ergosterol under anaerobic conditions (B).

To characterize enzymatic activity of the putative *C*. *reinhardtii* SQE, the coding region of the *CrSQE* gene was amplified from cDNAs of *C*. *reinhardtii* wild-type C-9 cells by RT-PCR. The product was cloned into a yeast expression vector pAUR123, containing the aureobasidin A-resistance gene *AUR1-C* as a selectable marker, under the control of the constitutive ADH1 promoter. The *S*. *cerevisiae ScERG1* ORF was cloned into the same vector as a positive control. The resultant expression plasmids and the empty vector were introduced into the *S*. *cerevisiae erg1* mutant KLN1 [30], and the corresponding transformants were isolated on aureobasidin A-containing selective Yeast extract-Peptone-Dextrose (YPD) medium. Three transformants harboring the *CrSQE* expression plasmid (CrSQE-1 to CrSQE-3) grew on YPD medium without ergosterol under aerobic conditions, as did two positive-control transformants, in contrast to the vector control, which did not grow (Fig. 2A). All of these transformants grew on YPD medium with ergosterol in anaerobic conditions (Fig. 2B). Ergosterol was detected both in the complementation lines expressing *CrSQE* and *ScERG1*, although large amounts of squalene still accumulated in the *CrSQE*-expressing line (S4 Fig.). These results indicated that the ergosterol biosynthetic pathway in the yeast *erg1* mutant could be reconstituted by heterologous expression of *CrSQE*, and CrSQE protein functions as SQE enzyme.

### Subcellular localization of CrSQS and CrSQE

**Fig 3:**
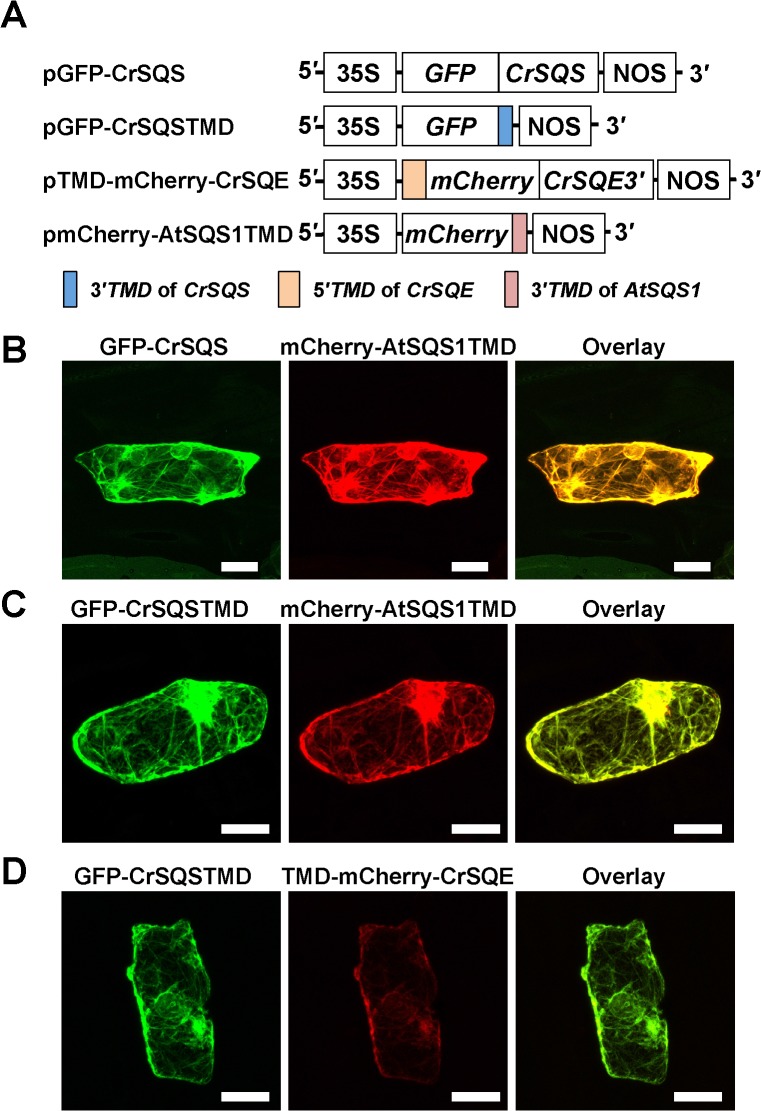
Subcellular localization of CrSQS and CrSQE in onion epidermal cells. A) Schematic diagrams of expression plasmids for GFP- and mCherry-fusion proteins. pGEP-CrSQS: GFP was fused at the C terminus to full-length CrSQS. pGFP-CrSQSTMD: GFP was fused at the C terminus to the C-terminal 118 residues of CrSQS including two predicted transmembrane domains (TMDs). pTMD-mCherry-CrSQE: mCherry was fused at its N terminus to the N-terminal 100 residues of CrSQE including a predicted TMD and at its C terminus to the C-terminal 423 residues of CrSQE including two predicted TMDs. pmCherry-AtSQS1TMD: mCherry was fused at the C-terminus to the C-terminal 67 residues of *A*. *thaliana* SQS1 including a predicted TMD, which had been shown to be localized in the endoplasmic reticulum [22]. 35S, cauliflower mosaic virus 35S promoter; NOS, terminator of nopaline synthase. B) The fusion proteins were expressed transiently in onion epidermal cells by particle bombardment. Fluorescence was observed using a laser scanning confocal microscope. Bars = 50 μM

To examine the subcellular localization of CrSQS, we expressed CrSQS and its C-terminal two TMDs as green fluorescent protein (GFP)-fusion proteins in onion epidermal cells (Fig. 3A). Each expression plasmid was co-transformed with an expression plasmid of mCherry-AtSQS1-TMD as an endoplasmic reticulum (ER)-localized marker [22]. The fluorescence images of both GFP-CrSQS and GFP-CrSQSTMD proteins were completely coincident with that of mCherry-AtSQS1-TMD protein in onion epidermal cells (Fig. 3B and 3C), suggesting that CrSQS is an ER-localized protein and its C-terminal TMDs function as ER-localization signal similarly to the C-terminal TMD in AtSQS1 protein [21,22].

To analyze subcellular localization of the CrSQE protein, mCherry protein fused at its N terminus to the N-terminal 100 residues of CrSQE containing the N-terminal TMD and at its C terminus to the C-terminal 423 residues of CrSQE containing the two C-terminal TMDs (TMD-mCherry-CrSQE protein) was expressed with GFP-CrSQSTMD protein in onion epidermal cells. The fluorescence images of TMD-mCherry-CrSQE protein were completely coincident with that of GFP-CrSQSTMD protein in onion epidermal cells (Fig. 3D), suggesting that CrSQE is also ER-localized.

### Overexpression of *CrSQS* gene in *C*. *reinhardtii*

**Fig 4:**
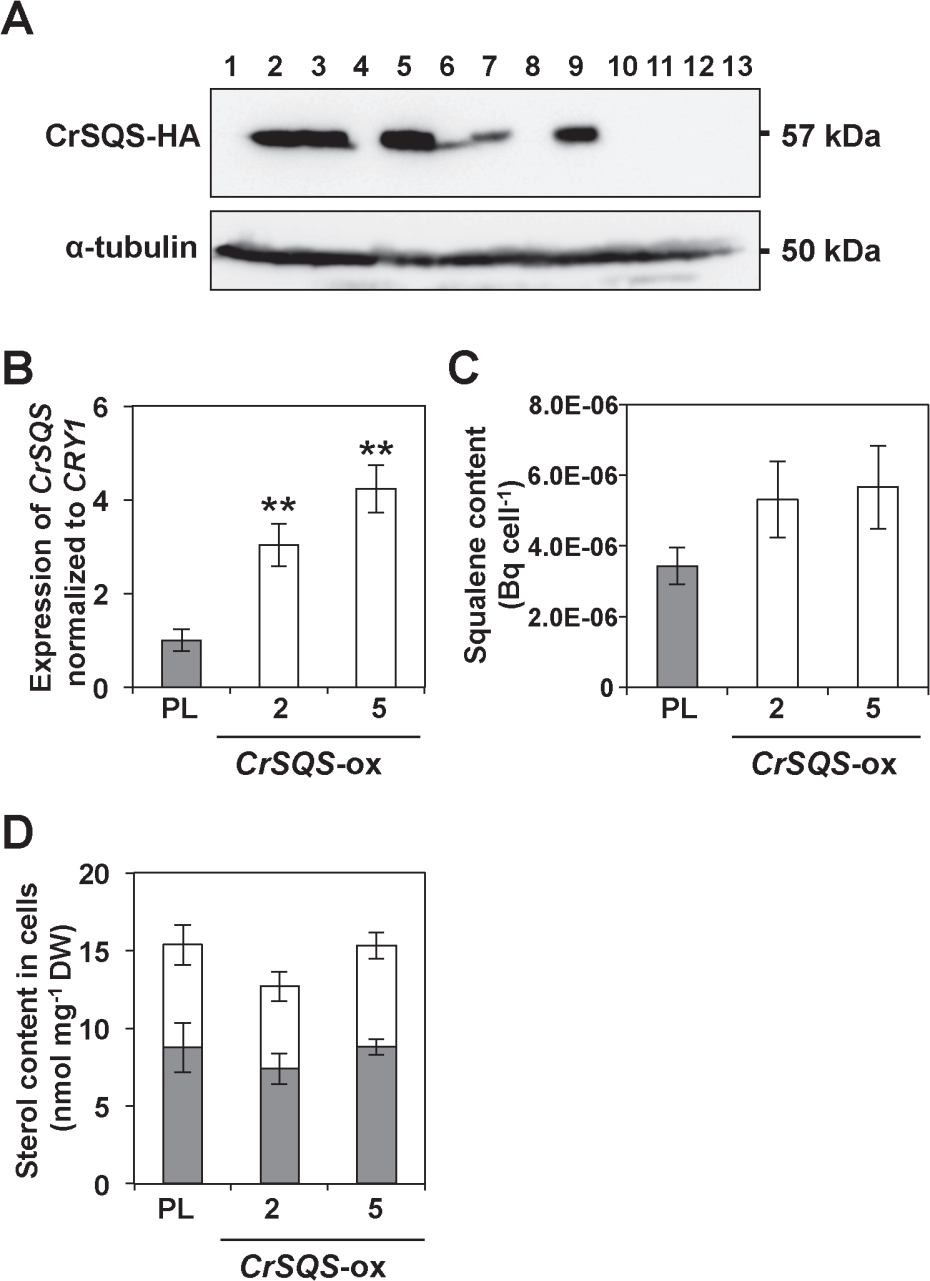
Overexpression of *CrSQS* gene in *C*. *reinhardtii* line UVM4. A) Immunoblotting analysis with antibodies against the HA epitope tag and α-tubulin in transgenic UVM4 lines harboring a *CrSQS*-expression plasmid. B) qRT-PCR analysis of *CrSQS* gene expression in a parental line (PL) UVM4 and two overexpression lines, *CrSQS*-ox-2 and *CrSQS*-ox-5. Expression of each gene was normalized to *CRY1*, which encodes ribosomal protein S14 [43]. C) Incorporation of (1–^14^C) FPP into squalene in each line. D) Content of ergosterol (grey bars) and putative 7-dehydroporiferasterol (open bars) in each line. Asterisks above the bars indicate significant differences (**p < 0.01). DW; cell dry weight. ND; not detected. Data in all experiments indicate mean value ± SD from three biological replicates.

To characterize the *in vivo* function of *CrSQS* in the squalene and sterol biosynthetic pathway, *CrSQS* was overexpressed in *C*. *reinhardtii* cells. A plasmid for overexpression of *CrSQS* (*CrSQS*-ox), comprising the genomic sequence of *CrSQS* coding region under the regulation of the constitutive *PsaD* promoter was expressed in an UVM4 mutant strain, which was suitable for the stable and effective expression of exogenous genes [32]. Two lines of transformants, *CrSQS*-ox-2 and *CrSQS*-ox-5, which showed positive signals at 57 kDa in the immunoblotting analysis using an anti-HA antibody, were used for further analysis (Fig. 4A). Total expression level of *CrSQS* from endogenous and exogenous loci was estimated by qRT-PCR (Fig. 4B). In the *CrSQS*-ox lines, expression levels of *CrSQS* were increased 3- to 4-fold compared with that in the parental line. Squalene synthesizing activity from (1–^14^C) FPP for 24 h in the *CrSQS*-ox cell cultures was 1.6- to 1.7-fold higher than that in the parental cells (Fig. 4C).

However, squalene was not detected in the two *CrSQS*-ox lines or parental line UVM4. The amounts of two major sterols, ergosterol and putative 7-dehydroporiferasterol, in all *CrSQS*-ox lines, were not changed significantly from those in UVM4 cells (Fig. 4D). These results suggested that squalene did not accumulate significantly in *CrSQS*-ox cells, although conversion of ^14^C-labeled FPP to squalene was enhanced by overexpression of *CrSQS*.

### Effect of SQE inhibitor terbinafine on cell viability and biosynthesis of squalene and sterols

**Fig 5:**
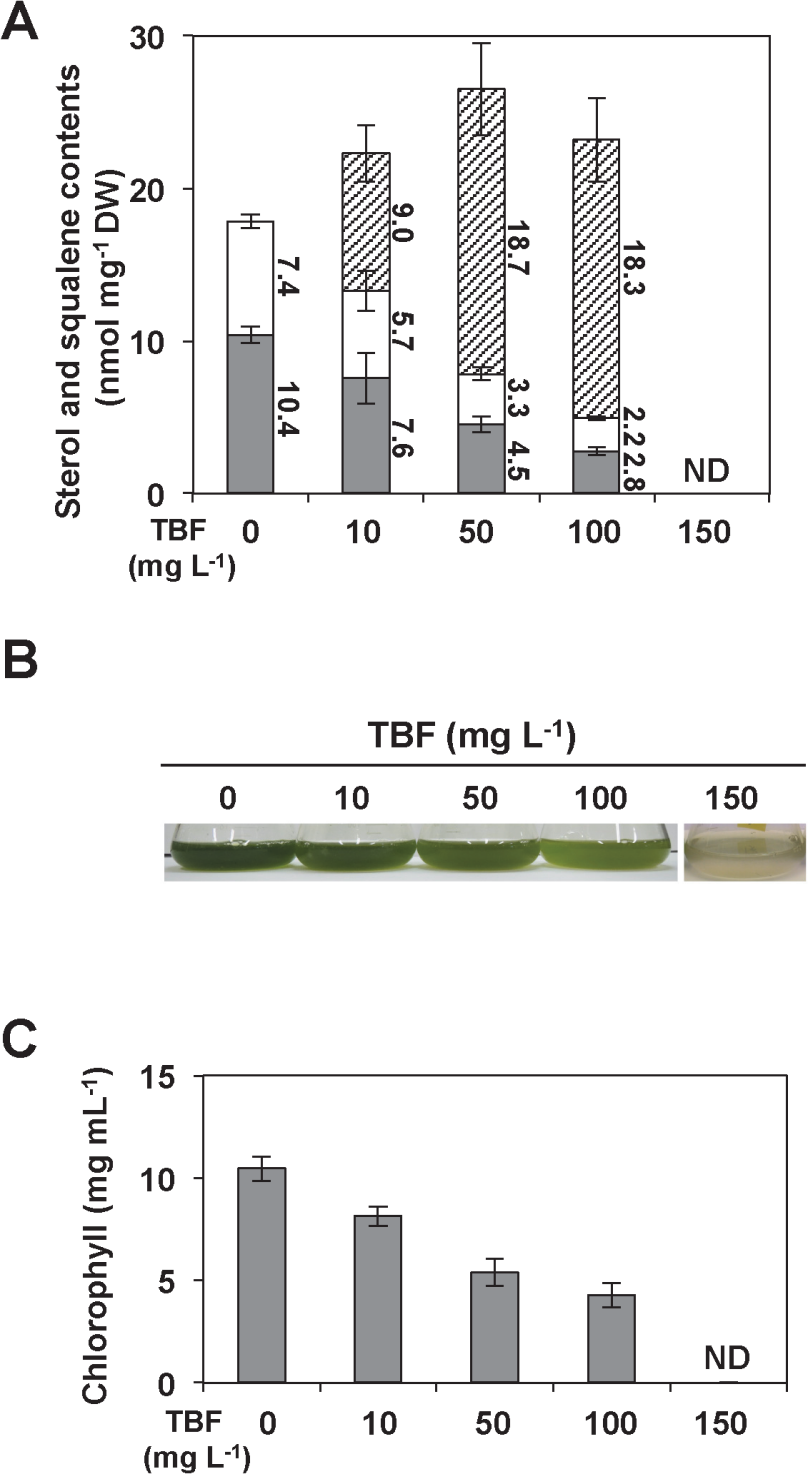
Effects of terbinafine (TBF) on squalene and sterol biosynthesis and cell viability. A) Contents of ergosterol (grey bars), putative 7-dehydroporiferasterol (open bars) and squalene (hatched bars) in the cells treated with different concentrations of TBF. DW; cell dry weight. B) Images of cultures treated with TBF. C) Chlorophyll content in cells treated with TBF. Data in all experiments are mean value ± SD from three biological replicates.

To verify the contribution of SQE activity to downstream sterol biosynthesis and the influence of SQE inhibition on the accumulation of squalene, dose-dependent inhibition of SQE activity was evaluated by addition of 10 to 150 mg L^–1^ terbinafine (TBF) [6,33–34] to cultures of UVM4 cells. The amount of squalene was increased by TBF treatment dose dependently, and reached 18.7 nmol mg^–1^ dry weight (DW) in the presence of 50 mg L^–1^ TBF (Fig. 5A). In contrast, the total amounts of ergosterol and putative 7-dehydroporiferasterol, which are downstream metabolites of the SQE reaction, were reduced by 72% in cells treated with 100 mg L^–1^ TBF compared with a TBF-untreated control (Fig. 5A). With a higher concentration of TBF of 150 mg L^–1^, cells were bleached (Fig. 5B and 5C) and squalene and sterols were not detected in the cell extracts (Fig. 5A), indicating that SQE activity was indispensable for sterol biosynthesis and cell survival.

### Knockdown of *CrSQE* in the UVM4 line

**Fig 6:**
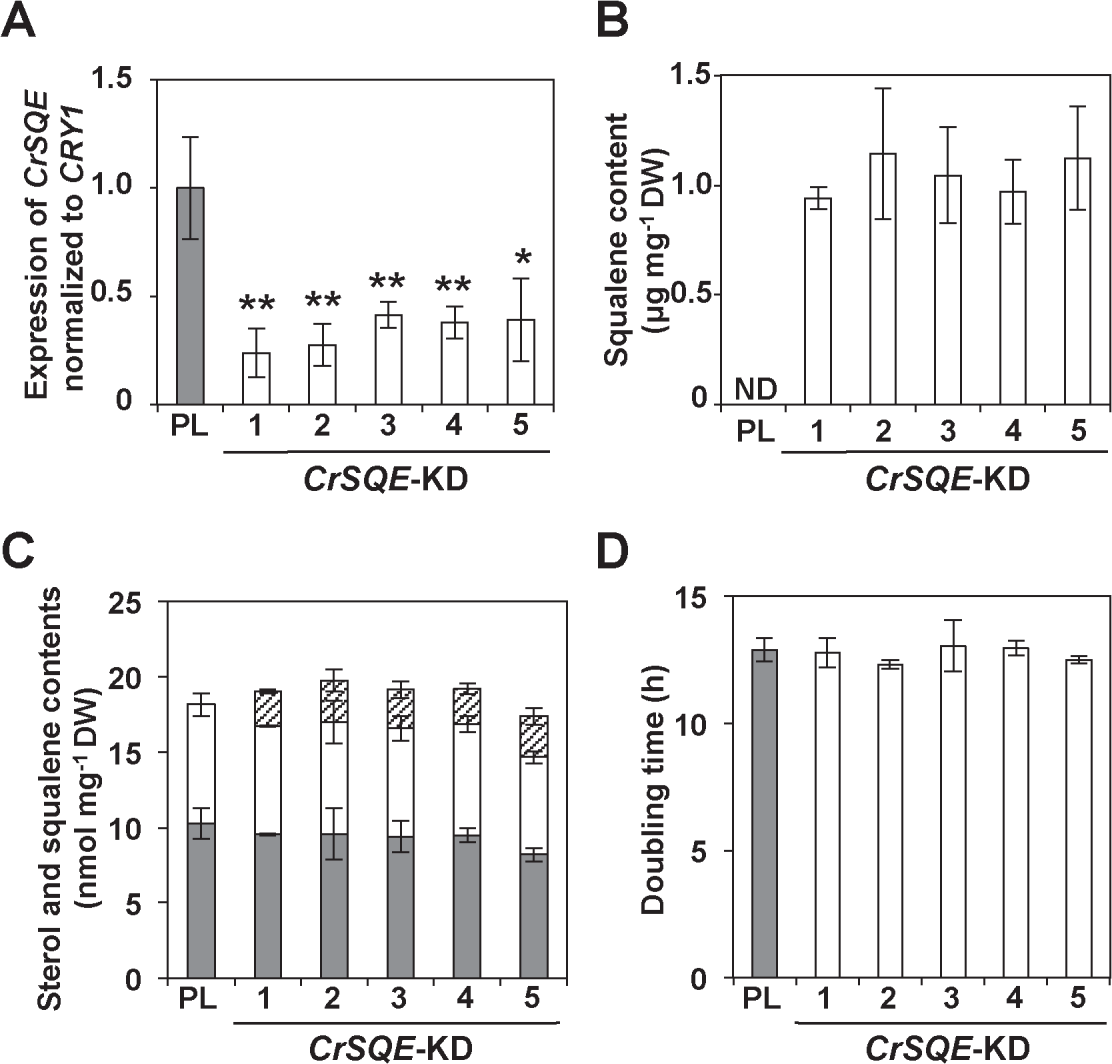
Knockdown of *CrSQE* gene in parental line (PL) UVM4. PL UVM4 and five knockdown lines (SQEKD-1 to SQEKD-5) were analyzed. A) qRT-PCR analysis of the *CrSQE* gene expression of each gene was normalized to that of the *CRY1* gene. Asterisks above the bars indicate significant differences (*p < 0.05, **p < 0.01). B) Squalene content was measured in each line. DW; cell dry weight. ND; not detected. C) Contents of ergosterol (grey bars), putative 7-dehydroporiferasterol (open bars) and squalene (hatched bars) were measured in each line. D) Doubling time in each line was calculated by measuring absorbance at 730 nm. Data in all experiments indicate mean value ± SD from three biological replicates.

To determine the functional importance of *CrSQE* in squalene and sterol biosynthesis, the expression of *CrSQE* was suppressed in UVM4 cells by expression of an artificial microRNA (amiRNA) [35]. Twenty-two out of 30 transformants were found to carry the *CrSQE*-KD plasmid by genomic PCR. The transcript level of *CrSQE* was significantly reduced by 59–76% in the five independent *CrSQE*-KD lines compared with that in the parental UVM4 line (Fig. 6A). In these lines, 0.9–1.1 μg (2.3–2.7 nmol) of squalene mg^–1^ cell DW accumulated (Fig. 6B and S5 Fig.). Unexpectedly, the amount of squalene in *CrSQE*-KD cells was 12–15% of that in parental UVM4 cells treated with 50 mg L^–1^ TBF. The amounts of ergosterol and putative 7-dehydroporiferasterol were unchanged among all *CrSQE*-KD lines and the parental UVM4 line (Fig. 6C), and no growth inhibition was observed in any of the *CrSQE*-KD lines (Fig. 6D).

To evaluate the effects of *CrSQE*-KD on gene expression involved in the MEP pathway, mRNA levels of 11 genes putatively encoding: 1-deoxy-D-xylulose 5-phosphate synthase (CrDXS), 1-deoxy-D-xylulose 5-phosphate reductoisomerase (CrDXR), 2-C-methyl-D-erythriol 4-phosphate cytidyltransferase (CrCMS), 4-diphosphocytidyl-2-C-methyl-D-erythritol 2-phosphate kinase (CrCMK), 2-C-methyl-D-erythritol 2,4-cyclodiphosphate synthase (CrMECPS), 4-hydroxy-3-methylbut-2-en-1-yl diphosphate synthase (CrHDS), isopentenyl diphosphate synthase (CrIDS), isopentenyl diphosphate isomerase (CrIDI), geranyl pyrophosphate synthase (CrGSP), farnesyl diphosphate synthase (CrFPS) and CrSQS annotated in the Phytozome database [18,19] (S1 Table), were measured by qRT-PCR. Expression levels of only *CrCMK* in all *CrSQE*-KD lines were increased by 2.2- to 4.9-fold compared with that in the parental line (S6A Fig.). In contrast, similar changes in mRNA levels of the other 8 genes, except for *CrIDS* and *CrIDI*, were not seen among the *CrSQE*-KD lines and the UVM4 parental line (S6B Fig.). Amplification of cDNAs from *CrIDS* and *CrIDI* in the parental line and all the *CrSQE*-KD lines were too weak to analyze. These results suggested that *CrSQE*-KD up-regulated expression of the putative *CMK* gene at the mRNA level.

### Screening of *CrSQS*-ox–*CrSQE*-KD UVM4 lines and the accumulation of squalene and sterols

**Fig 7:**
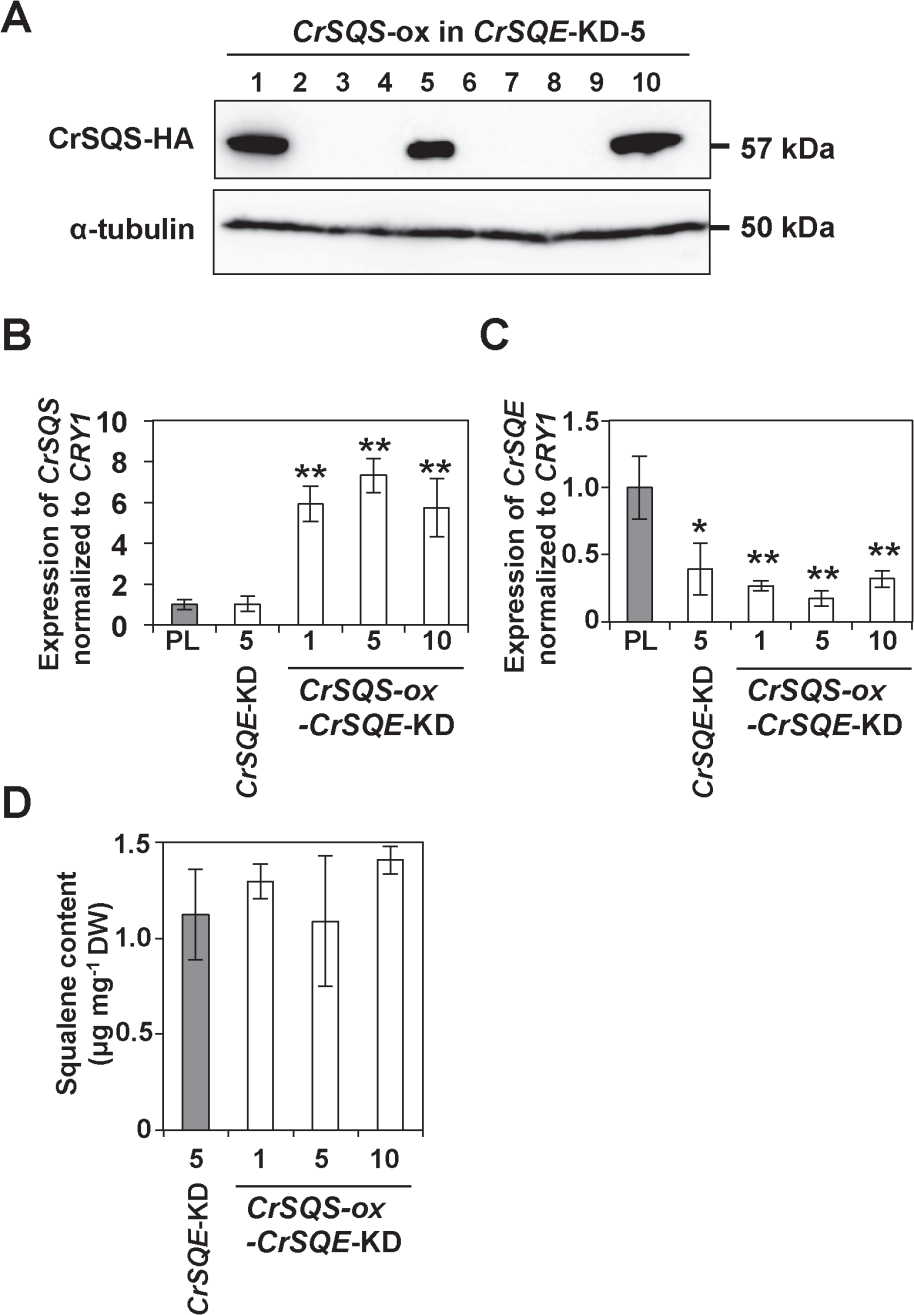
Overexpression of *CrSQS* gene and knockdown of *CrSQE* gene in parental line (PL) UVM4. A) Immunoblotting analysis with antibodies against the HA epitope tag and α-tubulin in the *CrSQE*-KD-5 lines harboring a *CrSQS*-expression plasmid. qRT-PCR analysis of the *CrSQS* (B) and *CrSQE* (C) genes in each line. Expression of each gene was normalized to that of the *CRY1* gene. D) Squalene content of the each line. DW; cell dry weight. Data in all experiments indicate mean value ± SD from three biological replicates.

To evaluate the synergistic effect on squalene accumulation by *CrSQS*-ox and *CrSQE*-KD in a single line, we transformed the *CrSQE*-KD-5 with the *CrSQS*-ox plasmid. The *CrSQS*-ox and *CrSQE*-KD lines were screened by immunoblotting analysis using anti-HA-tag antibody (Fig. 7A). Positive signals were detected in 3 out of 10 transformants, designated *CrSQS*-ox-*CrSQE*-KD-1, *CrSQS*-ox-*CrSQE*-KD-5 and *CrSQS*-ox-*CrSQE*-KD-10. As expected, the expression levels of *CrSQS* in these strains were 5.7- to 7.3-fold higher than those in the UVM4 and *CrSQE*-KD-5 lines (Fig. 7B). The expression levels of *CrSQE* were similar to those in the *CrSQE*-KD-5 line and were reduced by 68–82% compared with that in the UVM4 line (Fig. 7C). However, the amount of squalene was not changed from that in the *CrSQE*-KD-5 line (Fig. 7D), indicating that *CrSQS*-ox did not enhance the production and accumulation of squalene.

## Discussion

We isolated *CrSQS* and *CrSQE*, which are responsible for squalene synthesis and metabolism, from the model green alga *C*. *reinhardtii*, and demonstrated their enzymatic activities. Furthermore, analyses of transgenic *C*. *reinhardtii* indicated that squalene, which was undetectable in the parental strain, accumulated to approximately 0.1% (w/w) of cell DW by a 59–76% reduction in *CrSQE* expression in *CrSQE*-KD lines. As well as the levels of sterols, which are downstream products of squalene in the metabolic pathway, did not decrease significantly by suppression of *CrSQE* expression, growth of the *CrSQE*-KD lines did not differ significantly from that of the parental strain. These results can be explained by one of following hypotheses; (1) a sufficient amount of 2,3-oxidesqualene for sterol production could be supplied by the remaining CrSQE protein in the *CrSQE*-KD lines; (2) another gene(s) encoding a SQE could exist in addition to *CrSQE* in the *C*. *reinhardtii* genome, and its expression may not be suppressed in the *CrSQE*-KD lines; and (3) *C*. *reinhardtii* may have unknown bypass pathways for the SQE reaction for sterol supply. Based on the results of treatment with a SQE inhibitor on the parental UVM4 strain, we examined these hypotheses. Treatment with TBF decreased the amount of sterols and increased squalene accumulation in an dose-dependent manner. The amount of squalene in UVM4 cells treated with 50 mg L^–1^ TBF was 7- to 8-fold larger than that in *CrSQE*-KD lines. The chlorophyll content of the cells decreased dose dependently after TBF treatment, and cells were finally bleached in the presence of 150 mg L^–1^ TBF. These results indicated that TBF-sensitive SQE activity was indispensable for sterol biosynthesis and cell survival in algal cells. Based on these results and the fact that CrSQE had high levels of sequence similarity with the sequences of TBF-sensitive SQEs, such as ScERG1 [34], it was considered that the *CrSQE*-KD lines retained sufficient CrSQE activity to supply 2,3-oxidesqualene for downstream sterol production, although we could not completely exclude the second and third hypotheses. For accumulation of additional amounts of squalene, inducible-promoter-mediated transient KD of *CrSQE* could be effective.

On the other hand, we tried to increase the amount of squalene in cells by overexpression of *CrSQS*. However, the amount of squalene in *CrSQS*-ox lines was not increased compared with in parental cells, and the amounts of sterols were also unchanged between *CrSQS*-ox cells and the parental cells. Uptake experiments of ^14^C-labeled FPP suggested that the conversion of FPP to squalene was enhanced in *CrSQS*-ox lines. Therefore, the following possibilities could be considered as the reason why *CrSQS*-ox did not influence squalene accumulation; (1) the amount of FPP supplied via the MEP pathway was not enough for over accumulation of squalene, (2) an excess of squalene was converted rapidly to sterol intermediate(s) whose structure might not be suited for detection by gas chromatography analysis, (3) relatively insufficient increase of the accumulated level of CrSQS protein, although we have detected a significant level of increase in CrSQS mRNA by qRT-PCR, (4) the insufficient activity of the transgenic CrSQS protein with HA-tag at the C-terminal end, which may interfere its anchoring to the ER membrane, or (5) whose *K*_*m*_ value may be much higher than the concentration of intracellular FPP, (6) other types of SQS proteins may be operating to generate squalene *in vivo*, although the introduced by CrSQS was shown to have SQS activity in vitro, (cf. Botryococcene synthases showing a high level of sequence similarity with SQS did not have SQS activity in *B*. *braunii* [36]. In order to examine these possibilities, further investigation is necessary to reveal the reason why the overexpression of CrSQS was less effective on squalene accumulation.

Heterologous expression analysis in onion epidermal cells suggested that CrSQS and CrSQE were ER-localized proteins. *A*. *thaliana* AtSQS1 has also been reported as an ER-localized protein [22]. A predicted TMD at the C-terminus of AtSQS1 is necessary for localization to the ER [22]. Fungal and animal SQS proteins also have predicted C-terminal TMDs, which are thought to anchor the enzyme to the ER membrane [23]. CrSQS had two predicted TMDs at its C-terminus. A fusion protein of the C-terminal region including the TMDs with GFP showed an ER-localized pattern, indicating that the C-terminal TMDs of CrSQS play an important role in ER localization. In contrast to other SQS proteins that have one TMD at the C-terminus, two TMDs were predicted in the CrSQS sequence by the SOSUI program. The region where the first TMD was predicted in CrSQS was not predicted as a TMD in other SQS sequences. Because the region including both TMDs was cloned in the pGFP-CrSQSTMD plasmid in this study, further examination will be necessary to reveal the effect of each TMD on ER localization. These results suggested that the TMDs of CrSQE play an important role in ER localization, as in the case of CrSQS. The subcellular ER- and lipid droplet-localization of budding yeast SQE (ScERG1) has been reported by means of expression of a GFP fusion protein [37] and immunostaining [38]. Two TMDs in N- and C-termini were predicted in the ScERG1 sequence by the SOSUI program. However, the roles of the TMDs in the subcellular localization of SQE have not been clarified in any other organisms. In land plants, sterol biosynthesis downstream of squalene is predicted to be occurred in the ER [1]. These results suggested that the first steps catalyzed by CrSQS and CrSQE are also located in the ER in microalgal cells.

The mRNA level of a putative *CrCMK* gene was upregulated in all the *CrSQE*-KD lines. CMK is an enzyme that functions in the middle step of the MEP pathway and converts 4-diphosphocytidyl-2C-methyl-D-erythritol to 4-diphosphocytidyl-2-C-methyl-D-erythritol 2-phosphate. Only one gene showing sequence similarity to characterized *CMKs* in other organisms was annotated in the *C*. *reinhardtii* genome database in Phytozome (*CrCMK*; *Cre16*.*g679669*) [18,19]. In *A*. *thaliana*, regulation of enzymes in the MEP pathway has been reported [39]. In all the mutants of *DXR*, *CMS*, *HDS* and *HDR*, the protein level of *DXS*, which catalyzes the first step in the MEP pathway, was higher than that in the wild type. In contrast, the mRNA level of DXS in the mutants was lower than that in the wild type, suggesting the presence of post-transcriptional regulation of the DXS protein [39]. However, the mRNA and protein levels of CMK in the mutants were lower than those in the wild type. These results indicated that KD of CrSQE generated a change in gene expression related to the upstream MEP pathway. In *CrSQE*-KD lines, similar changes in mRNA levels were not observed in other genes in the MEP pathway, but some of them might be regulated post-transcriptionally.

Based on the results of this study in transgenic microalgae, we conclude that the partial suppression of *CrSQE* expression is an effective strategy to increase squalene accumulation. A feasible strategy to accumulate still higher levels of squalene in cells is to increase the amount of FPP supplied through enhancement of the MEP pathway by overexpression of *CrDXS*, *CrDXR*, and *CrHDS*, which encode candidates of rate-limiting enzymes in land plants [10], and other genes involved in the MEP pathway in a background of *CrSQE*-KD and/or *CrSQS*-ox transgenic lines.

## Material and Methods

### Strains and culture conditions

For isolation of genomic DNA and total RNA in *C*. *reinhardtii*, a wild-type C-9 strain (mt^–^) was grown for 2 days in 50 mL Tris-Acetate-Phosphate (TAP) medium [40] at 25°C under continuous light illumination at 50 μmol photons m^–2^ s^–1^ on an orbital shaker at 100 rpm. A mutant strain UVM4 [32] that efficiently expresses transgenes was used for functional analysis of *CrSQS* and *CrSQE*. For metabolic and expression analysis, UVM4 and transgenic lines were cultured for 2 days under the same conditions as the C-9 culture. For the inhibitor studies, the UVM4 cells were cultured for 1 day in 50 mL TAP liquid medium to OD_730_ 0.1, and a SQE-inhibitor, TBF (Sigma-Aldrich, St. Louis, MO), was added in the culture to a final concentration of 0, 10, 100, 150, and 300 mM, respectively, and then further incubated for 1 day.

### Quantitative (q)RT-PCR

qRT-PCR was performed using SYBR Premix Ex Taq GC (Takara Bio, Shiga, Japan) and a LightCycler 480 Instrument (Roche, Basel, Switzerland) as described previously [41]. *CRY1* encoding ribosomal protein S14 [42] was used as an internal control [43]. The primers used for qRT-PCR are listed in S1 Table.

### Southern blotting analysis

Genomic DNA was extracted from C-9 cells using a DNeasy Plant Mini Kit (Qiagen, KJ Venlo, Netherlands). Five μg of genomic DNA digested with restriction enzymes was separated by electrophoresis, transferred to a nylon membrane (Biodyne B; Pall Corporation, Port Washington, NY), and hybridized using standard protocols [44]. For detection of *CrSQS* and *CrSQE*, their coding sequences were amplified by PCR with the primers for the construction of expression vectors for *C*. *reinhardtii* and yeast, respectively. The PCR products were then labeled with (*α*-^32^P) dCTP using Ex-Taq DNA polymerase (Takara Bio) and used as probes. Hybridization was performed using ExpressHyb hybridization solution (Clontech, Mountain View, CA) in accordance with the manufacturer’s instructions.

### Immunoblotting analysis

Immunoblotting analysis was performed as described previously [41,45]. The following antibodies were used: a horseradish peroxidase (HRP)-linked monoclonal anti-HA antibody (1: 2,000 dilution; Roche, clone 3F10) and anti-α-tubulin antibody (1:5,000 dilution; Sigma-Aldrich, clone B-5-1-2). HRP-conjugated goat anti-mouse IgG antibody (1:10,000 dilution; GE Healthcare, Buckinghamshire, United Kingdom) to detect α-tubulin was used as a secondary antibody. Tris-buffered saline with 0.05% Triton X-100 was used for the dilution of antibodies.

### Expression of recombinant CrSQS protein in *Escherichia coli*

A partial coding region of *CrSQS* cDNA (+1 to +1191) excluding the putative TMD was amplified using the primer set CrSQS-pET-F and CrSQS-pET-R. cDNAs from C-9 cells cultured for 2 days as described above were used as the template. Sequences of all primers for plasmid construction and transgenic analysis in this study are listed in S2 Table. PCR was carried out by PrimeScript GXL Polymerase (Takara Bio). The resultant PCR product was cloned into a pET-21b vector (Merck Millipore, Darmstadt, Germany) using an In-Fusion HD Cloning kit (Clontech). The recombinant His-tagged CrSQS protein was induced in *E*. *coli* strain BL21 (DE3) (Life Technologies, Carlsbad, CA) by the addition of 0.5 mM isopropylthio-*ß*-galactoside for 22°C for 6 h. The recombinant protein was extracted under anaerobic conditions using BugBuster Master Mix protein extraction reagents (Merck Millipore) and purified using a TALON Metal Affinity Resin (Clontech) in accordance with to the manufacturer’s instructions. Eluted fractions containing the recombinant protein were combined and dialyzed against phosphate-buffered saline buffer (pH 7.4) containing 100 mM sucrose, 5 mM dithiothreitol (DTT), and Complete Protease Inhibitor Cocktail (Roche) using a Slide-A-Lyzer Dialysis Cassette 3.5K (Thermo Fisher Scientific, Waltham, MA). The crude cell lysate and purified and CrSQS protein used for the enzyme assay. BL21 (DE3) cells harboring the empty pET-21b vector were cultured under the same induction condition, and the cell lysates used as a control.

### Assay for CrSQS activity

The enzymatic activity of the recombinant CrSQS protein was measured as described previously [22,46] with some modifications. The reaction mixture was contained in a total volume of 100 μL: 25 μM (1–^14^C) FPP (1.2 kBq; American Radiolabeled Chemicals, St. Louis, MO), 2.5 mM NADPH (Sigma-Aldrich), 50 mM glucose-6-phosphate (Sigma-Aldrich), 2U glucose-6-phosphate dehydrogenase (Sigma-Aldrich), 5 mM MgCl_2_ (or 2 mM MnCl_2_), 10 mM DTT, 50 mM potassium phosphate (pH 7.4), 1 mg mL^–1^ bovine serum albumin and 15 μg purified CrSQS (or 15 μg of crude cell lysate). The reaction mixture was incubated at 30°C for 1 h, and the reaction was stopped by the addition of 10 μL of a solution containing 0.4% v/v squalene, 1 M Na_2_EDTA (pH 9.2), and 1% v/v ethanol. The mixture was quickly placed in an ice bath and used directly for TLC analysis. For normal-phase TLC analysis of the reaction products, 10 μL of the reaction mixture was applied onto a silica gel 60G F_254_ plate (Merck Millipore). The plates were developed with cyclohexane:ethyl acetate (9:1, v/v). For reverse-phase TLC analysis, 20 μL of reaction mixture was applied onto silica gel 60 RP-18 F_254S_ plate (Merck Millipore), which was developed with acetone:water (19:1, v/v). After development, the plates were exposed and analyzed using a bio-image analyzer, Typhoon FLA7000 (GE Healthcare). Squalene standard (Sigma-Aldrich) were visualized by UV irradiation.

### Expression of *CrSQE* in the yeast KLN1 mutant

Functional identification of *CrSQE* was achieved by heterologous expression in the yeast *ScERG1*-defective mutant KLN1 (*MATα*, *erg1*::*URA3*, *leu2*, *ura3*, *trp*) [30]. The coding region of the *CrSQE* cDNA was amplified using primer set CrSQE-pAUR123-F and CrSQE-pAUR123-R. cDNAs from C-9 cells cultured for 2 days as described above were used as the template. PrimeScript GXL Polymerase (Takara Bio) was used for PCR. As a positive control, the *ScERG1* gene was amplified using primer set ScERG1-pAUR123-F and ScERG1-pAUR123-R. Genomic DNA from a yeast wild-type InvSc1 strain (Life Technologies) was used as a template. Each PCR product was cloned into a pAUR123 vector (Takara Bio). The resultant constructs, pAUR123-CrSQE and pAUR123-ScERG1, were used to transform the KLN1 mutant by the lithium acetate method [47]. Transformants were selected on YPD agar medium containing 1% yeast extract, 2% peptone, 2% dextrose and 2% agar with 0.5 μg mL^–1^ aureobasidin A (Takara Bio). Anaerobic conditions were achieved by culturing the yeast transformants in an anaerobic jar with AnaeroPack-Anaero (Mitsubishi Gas Chemical, Tokyo, Japan). Ergosterol was dissolved in Tween-80:ethanol (1:1, v/v) and supplied in YPD medium at a final concentration of 20 μg mL^–1^.

### Expression of GFP-CrSQS and mCherry-CrSQE fusion proteins in onion epidermal cells

A PCR fusion-based approach was applied to create reporter gene constructions for transient expression analysis in onion epidermal cells. All of the PCR reactions were done using PrimeScript GXL Polymerase (Takara Bio). Firstly, the sequences encoding the full-length *CrSQS* and *CrSQS-TMD* cDNAs were amplified using cDNAs from C-9 cells cultured for 2 days as described as templates and the primer sets sGFP-CrSQS-F and CrSQS-pBI221-R for full-length *CrSQS* cDNA, and sGFP-CrSQSTMD-F and CrSQS-pBI221-R for the *CrSQS-TMD* cDNA. The sequences encoding 5′- and 3′-partial fragments of *CrSQE* were amplified using pAUR123-CrSQE plasmid as a template and the primer set CrSQE-pBI221-F and SQE300-mCherry-R for the 5′-partial fragment and mCherry-CrSQE301-F and CrSQE-pBI221-R for the 3′-partial fragment. The sequence encoding *AtSQS1TMD* [22] was amplified using the primers mCherry-AtSQS1TMD-F and AtSQS1-pBI221-R and cDNAs from seedlings of wild-type *A*. *thaliana* Col-0 as a template. Translational start and stop codons are shown in bold letters (S2 Table).

Secondly, the coding sequence of the GFP gene was amplified using primer set sGFP-pBI221-F and sGFP-CrSQS-R for fusion with the full-length *CrSQS* fragment and sGFP-pBI221-F and sGFP-CrSQSTM-R for fusion with the *CrSQS-TMD* fragment. The coding sequence of the mCherry gene was amplified using primers SQE300-mCherry-F and mCherry-SQE301-R for fusion with the 5′- and 3′-partial fragments of *CrSQE* cDNA, and sGFP-pBI221-F and mCherry-AtSQS1TM-R for fusion with the *AtSQS1-TMD* cDNA fragment.

Finally, two amplicons for each construct were mixed and used as templates to fuse to each other by PCR with the primer pairs sGFP-pBI221-F and CrSQS-pBI221-R for fusion of GFP with the full-length *CrSQS* and *CrSQS-TMD* fragments; sGFP-pBI221-F and CrSQE-pBI221-R for fusion of mCherry with the 5′- and 3′-partial fragments of *CrSQE*; and sGFP-pBI221-F and AtSQS1-pBI221-R for fusion of mCherry with the *AtSQS1-TMD* fragment. The resultant fused fragments were cloned between *Xba*I and *Sac*I sites in a pBI221 expression vector (Clontech) using an In-Fusion cloning kit (Clontech). The resultant plasmids were transformed into onion epidermal cells by particle bombardment using a PDS-1000/He Biolistic Particle Delivery System (Bio-Rad, Hercules, CA) equipped with 1,100 psi rupture disks. After incubation in the dark at 22°C for 24 h, the fluorescence signals of each reporter protein was observed using a Leica TCS SP8 laser scanning confocal microscope (Leica, Solms, Germany) with excitation at 488 nm for GFP and 552 nm for mCherry fluorescence.

### Transgenic analysis in *C*. *reinhardtii*

For overexpression of *CrSQS*, its coding sequence was amplified using genomic DNA from C-9 cells as a template and primer set CrSQSox-F and CrSQSox-R. The resultant PCR product was cloned between the *PsaD* promoter [48] and a HA-tag in a pGenD-cHA-Hyg3 plasmid, which was constructed from a pGenD-cHA plasmid [49] by replacement of the *aphVIII* expression cassette with an *aph7"* expression cassette derived from a pHyg3 plasmid [50].

For amiRNA-mediated KD of *CrSQE*, two pairs of oligonucleotides, CrSQEKD-F and CrSQEKD-R, were designed as described previously [35] using the WMD3-Web MicorRNA Designer site (http://wmd3.weigelworld.org/). These oligonucleotides were annealed and cloned in a pChlamiRNA3-int vector [35]. The resultant constructs were transformed into the parental line UVM4 by an electroporation method [51]. Transformants were screened by PCR using primers PsaD-F and CrSQSox-02R for *CrSQS*-ox lines, and AmiRNAprec and pChlamiRNA3-R for *CrSQE*-KD lines.

### Measurements of squalene and sterol content

Squalene and sterols were extracted from the *C*. *reinhardtii* cells as described previously [14,15] with slight modifications. *C*. *reinhardtii* cells cultured in 50 mL TAP medium for 2 days (approximately 1×10^7^ cells mL^–1^) were dried and mixed with 20 mL of 1 mg mL^–1^ cholesterol (Sigma-Aldrich) as an internal control and saponified in 2 mL of 10% KOH containing 50% methanol for 30 min by sonication. After extraction with the same volume of hexane, the solvent was evaporated and the residue dissolved in 20 μL of chloroform. Then, 20 μL of a silylating reagent, N,O-bis(trimethylsilyl)trifluoroacetamide (Sigma-Aldrich) was added to the lipid samples and incubated at 80°C for 30 min. Derivatized samples were increased to a volume of 40 μL with chloroform. A 2 μL aliquot of the solution was analyzed using a gas chromatography (GC-4000; GL Science, Tokyo, Japan) equipped with an InertCap 5 capillary column (30 m × 0.25 mm, film thickness 0.25 mm, GL Science) and gas chromatography-mass spectrometry (GCMS-QP2010 Ultra; Shimadzu, Kyoto, Japan) equipped with an DB-5MS capillary column (30 m × 0.25 mm, film thickness 0.25 mm, Agilent Technologies, Santa Clara, CA), carrier gas: He (1 mL min^–1^), oven temperature: 150–300°C (increase rate 20°C min^–1^). The ionization voltage was 70 eV and scan range was 40–500 Da in GC-MS analysis. The squalene and ergosterol contents were calculated from the ratios of the peak areas of each standard (Sigma-Aldrich). The second largest peak among the endogenous sterols from *C*. *reinhardtii* wild-type cells was estimated as putative 7-dehydroporiferasterol by compared gas-chromatogram pattern (S5A Fig.) and mass fragmentation pattern of electron ionization (EI) in GC-MS analysis (S5B Fig.) with those in the reference [15]. The content of putative 7-dehydroporiferasterol was estimated based on the standard curve of authentic ergosterol.

### Squalene-synthesizing activity from ^14^C-FPP

After 2-days of incubation, cells were collected by centrifugation at 600 g for 5 min, and the obtained total 3×10^8^ cells were suspended in new TAP medium (3 mL) in a 30 mL Erlenmeyer flask fitted with a glass tube that contained a piece of folded filter paper impregnated with 0.1 mL 20% KOH in the center well to collect ^14^CO_2_, and were incubated with 7.4 kBq of (1–^14^C) FPP (2.22 GBq mmol^–1^, American Radiolabeled Chemicals) in the light (10.2 μmol photons m^–2^ s^–1^) for 24 h. The hydrophobic fraction was extracted from harvested cells by the method described previously [52]. The hydrophobic fraction was separated by TLC using cyclohexane/ethylacetate (9:1, v/v). The plate was sprayed with 0.01% (w/v) primulin (Tokyo Chemical Industry, Tokyo, Japan) in 80% (v/v) acetone, and the lipid spots were located using ultraviolet light. The ^14^C-labeled compounds on the TLC plates were detected using a Bio-Imaging Analyzer (Typhoon FLA7000, GE Healthcare).

## Supporting Information

S1 Fig Amino acid sequence alignments of CrSQS (A) and CrSQE (B) and related sequences. Sequences were aligned using the ClustalW [53] method. In the alignment of SQS proteins, the three conserved domains involved in catalysis [22] are underlined in blue. In the alignment of SQE proteins, highly conserved sequences containing the putative FAD-binding domain [26,46] are boxed in red. Predicted transmembrane domains are shown in yellow boxes in each sequence. Identical residues in least two sequences are shown with a black background. Similar residues are shown with a gray background. See S3 Table for accession numbers for each protein sequences. See S1 Text for sequence information of SQS and SQE as FASTA format data.
(TIF)

S2 Fig Southern blotting analysis of the *CrSQS* and *CrSQE* genes. Genomic DNA isolated from wild-type C-9 cells was digested with *Xho*I and *Bam*HI for detection of the *CrSQS* gene and *Hin*dIII and *Xho*I for detection of the *CrSQE* gene, and hybridized with a ^32^P-labeled DNA fragment from each ORF. No recognition sites for *Xho*I, *Bam*HI or *Hin*dIII occurred within the coding sequence of each gene. One recognition site for *Xho*I occurred within the coding sequence of *CrSQE*. The size of each restriction fragment detected is given on the right in kb.
(TIF)

S3 Fig Mg^2+^ and Mn^2+^ as co-factors in SQS enzymatic assays. The metal co-factor requirements were examined in enzymatic assays with CrSQS, and the products were analyzed by normal-phase (A) and reverse-phase (B) thin-layer chromatography. The different components included in the reaction mixtures are indicated at the top. Authentic (1–^14^C) farnesyl diphosphate (FPP) was loaded as a negative control. The positions of the origin, solvent front and authentic squalene are indicated on the left. Open triangles on the right indicate positions of signals from putative dehydrosqualene and 12-hydroxysqualene.
(TIF)

S4 Fig Squalene and sterol profiles in complementation lines of the KLN mutant expressing *CrSQE* and *ScERG1*. Complementation lines of the KLN mutant expressing *CrSQE* and *ScERG1* were established. Squalene and sterols were extracted from yeast cells harvested from YPD culture plates in aerobic conditions (Fig. 2A) and measured by gas chromatography analysis. 1, squalene; 2, cholesterol (internal standard); 3, ergosterol. Gas-chromatograms of each authentic standard were shown in the lower part.
(TIF)

S5 Fig Squalene and sterol profiles in *CrSQE*-KD-1 and parental UVM4 lines. A) Squalene and sterols were extracted from *C*. *reinhardtii* cells cultured in TAP liquid medium for 2 day as in Fig. 6, and measured by gas chromatography analysis. Gas-chromatograms of each authentic standard were shown in lower panels. 1, squalene; 2, cholesterol (internal standard); 3, ergosterol. The peak 4 was estimated to be putative 7-dehydroporiferasterol. B) Mass spectrum of peak 4 measured by gas chromatography-mass spectrometry analysis.
(TIF)

S6 Fig Expression analysis of nine putative genes related to the methylerythritol phosphate (MEP) pathway. Expression levels of genes putatively involved with the MEP pathway were examined in CrSQE-knockdown (KD) lines. Nine genes encoding putative enzymes in the MEP pathway were found in the Phytozome database. Among them, the expression level of a putative *CMK* gene was examined in all five *CrSQE*-KD lines (A). Expression levels of the other eight genes were examined in two *CrSQE*-KD lines, *CrSQE*-KD-2 and *CrSQE*-KD-4, as representative lines (B). Primer sequences, each gene ID in Phytozome and their encoded enzyme names are listed in S1 Table. Expression of each gene was normalized to that of *CRY1*. Data in all experiments indicate mean value ± SD from three biological replicates. Asterisks above the bars indicate significant differences (*p < 0.05, **p < 0.01).
(TIF)

S1 Table Primer sequences for qRT-PCR. (DOC)

S2 Table Primer sequences for transgenic analysis. (DOC)

S3 Table Accession numbers of SQS and SQE sequences used for alignment in S1 Fig.
(DOC)

S1 Text Amino acid sequences of SQS and SQE used for alignment in S1 Fig. and listed in S3 Table. (TXT)
