# Supplementary figures and images for: Accumulation of Squalene in a Microalga Chlamydomonas reinhardtii by Genetic Modification of Squalene Synthase and Squalene Epoxidase Genes
Source: PLoS One. 2015 Mar 12;10(3):e0120446. doi: 10.1371/journal.pone.0120446 (PMC4357444; doi:10.1371/journal.pone.0120446)

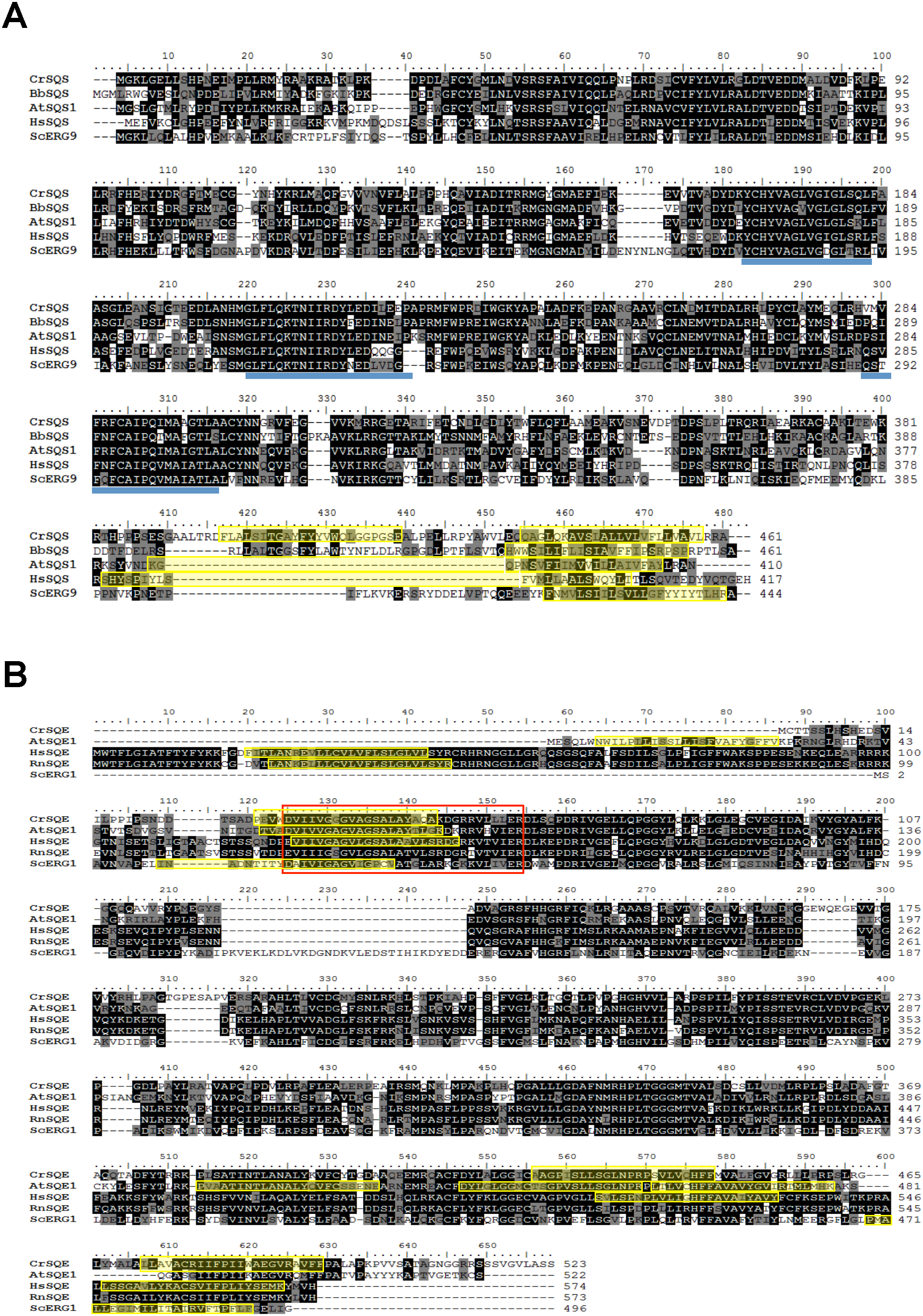

Supplement: S1 Fig — Sequences were aligned using the ClustalW [53] method. In the alignment of SQS proteins, the three conserved domains involved in catalysis [22] are underlined in blue. In the alignment of SQE proteins, highly conserved sequences containing the putative FAD-binding domain [26,46] are boxed in red. Predicted transmembrane domains are shown in yellow boxes in each sequence. Identical residues in least two sequences are shown with a black background. Similar residues are shown with a gray background. See S3 Table for accession numbers for each protein sequences. See S1 Text for sequence information of SQS and SQE as FASTA format data. (TIF) [file pone.0120446.s001.tif]

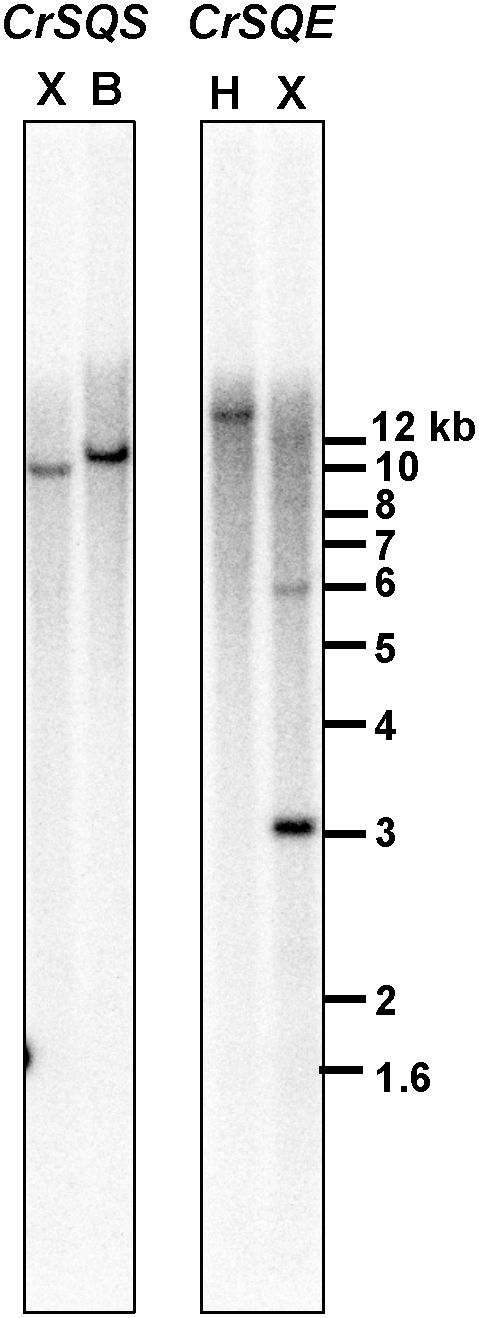

Supplement: S2 Fig — Genomic DNA isolated from wild-type C-9 cells was digested with XhoI and BamHI for detection of the CrSQS gene and HindIII and XhoI for detection of the CrSQE gene, and hybridized with a 32P-labeled DNA fragment from each ORF. No recognition sites for XhoI, BamHI or HindIII occurred within the coding sequence of each gene. One recognition site for XhoI occurred within the coding sequence of CrSQE. The size of each restriction fragment detected is given on the right in kb. (TIF) [file pone.0120446.s002.tif]

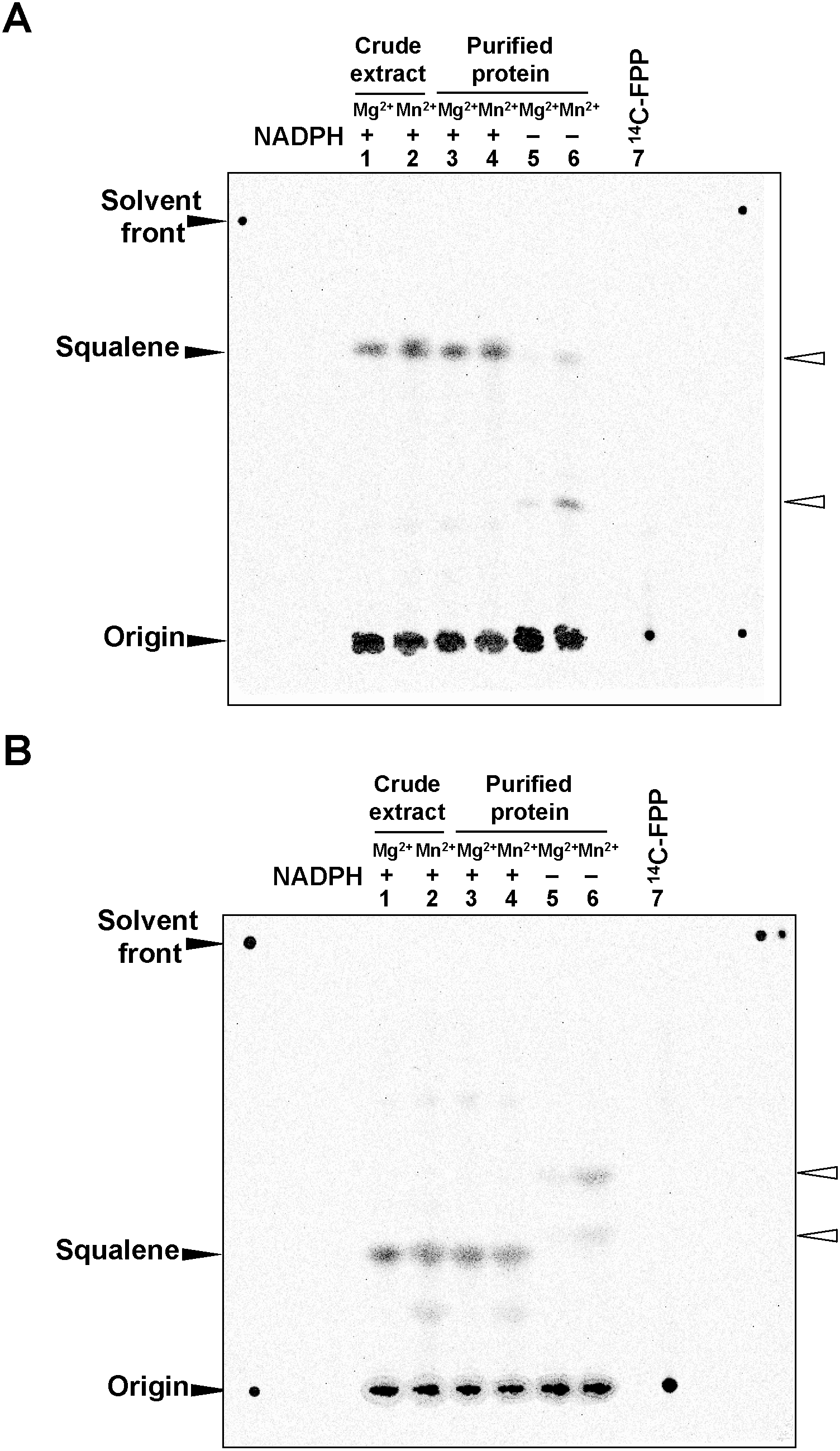

Supplement: S3 Fig — The metal co-factor requirements were examined in enzymatic assays with CrSQS, and the products were analyzed by normal-phase (A) and reverse-phase (B) thin-layer chromatography. The different components included in the reaction mixtures are indicated at the top. Authentic (1–14C) farnesyl diphosphate (FPP) was loaded as a negative control. The positions of the origin, solvent front and authentic squalene are indicated on the left. Open triangles on the right indicate positions of signals from putative dehydrosqualene and 12-hydroxysqualene. (TIF) [file pone.0120446.s003.tif]

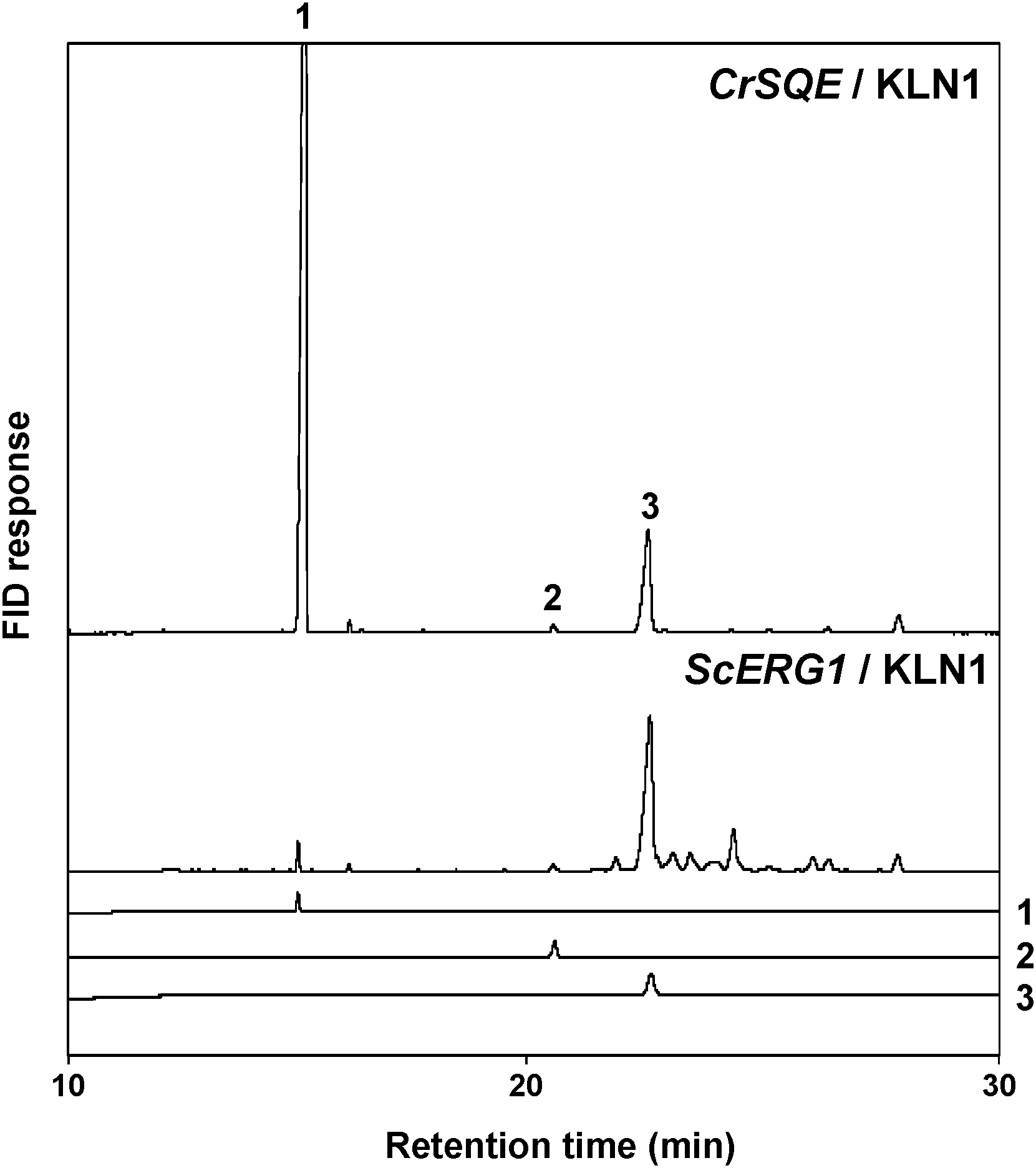

Supplement: S4 Fig — Complementation lines of the KLN mutant expressing CrSQE and ScERG1 were established. Squalene and sterols were extracted from yeast cells harvested from YPD culture plates in aerobic conditions (Fig. 2A) and measured by gas chromatography analysis. 1, squalene; 2, cholesterol (internal standard); 3, ergosterol. Gas-chromatograms of each authentic standard were shown in the lower part. (TIF) [file pone.0120446.s004.tif]

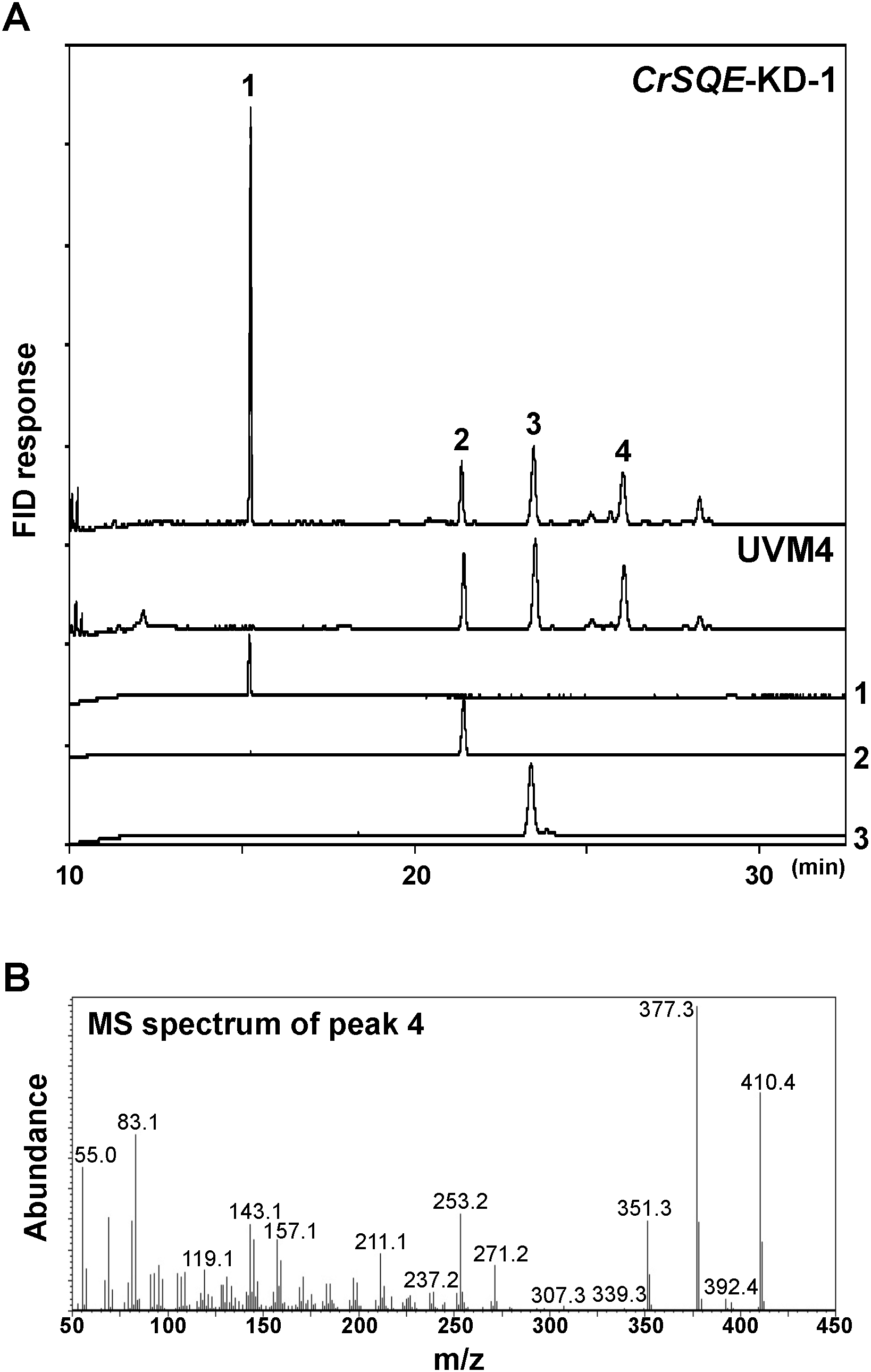

Supplement: S5 Fig — A) Squalene and sterols were extracted from C. reinhardtii cells cultured in TAP liquid medium for 2 day as in Fig. 6, and measured by gas chromatography analysis. Gas-chromatograms of each authentic standard were shown in lower panels. 1, squalene; 2, cholesterol (internal standard); 3, ergosterol. The peak 4 was estimated to be putative 7-dehydroporiferasterol. B) Mass spectrum of peak 4 measured by gas chromatography-mass spectrometry analysis. (TIF) [file pone.0120446.s005.tif]

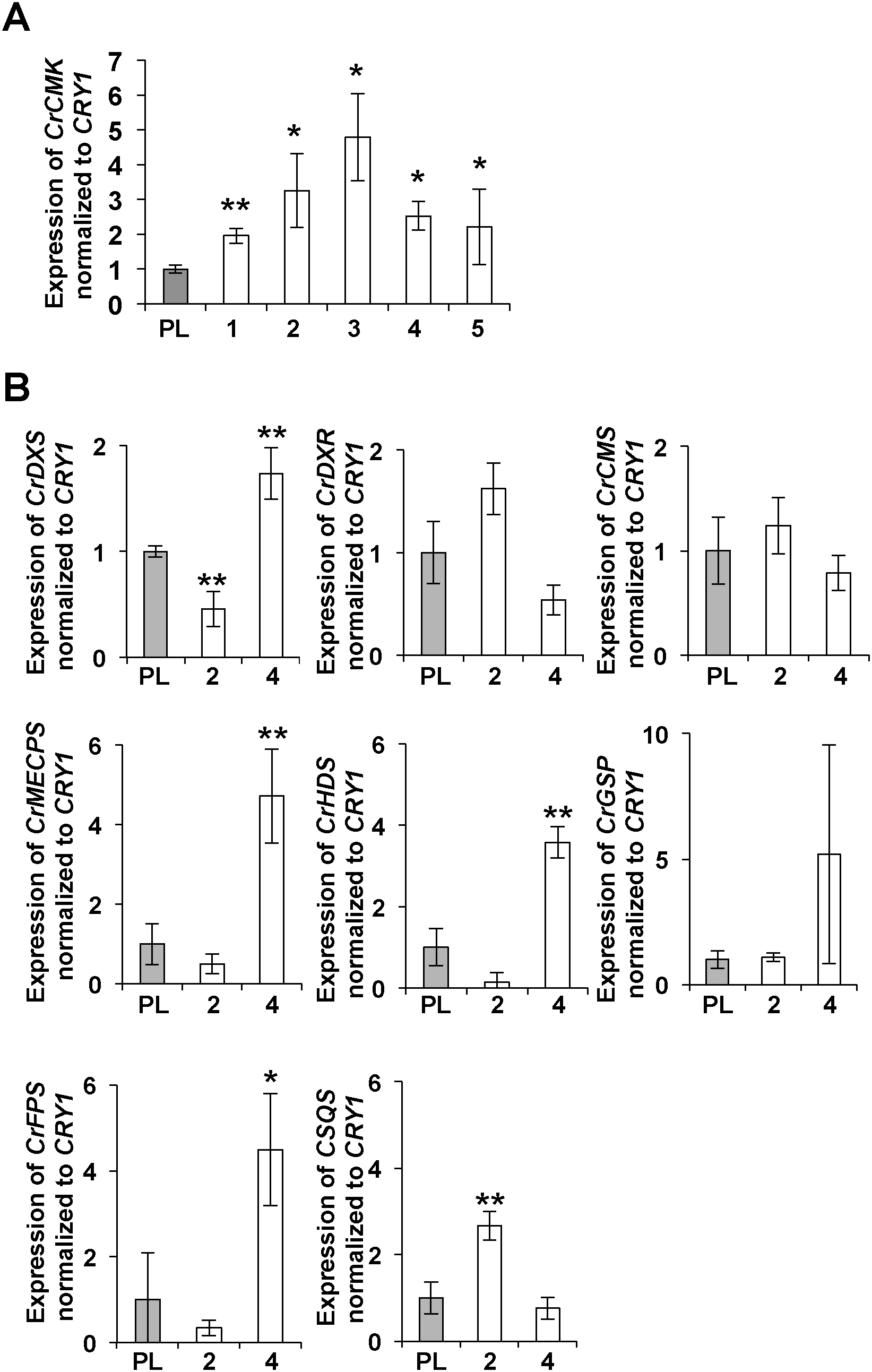

Supplement: S6 Fig — Expression levels of genes putatively involved with the MEP pathway were examined in CrSQE-knockdown (KD) lines. Nine genes encoding putative enzymes in the MEP pathway were found in the Phytozome database. Among them, the expression level of a putative CMK gene was examined in all five CrSQE-KD lines (A). Expression levels of the other eight genes were examined in two CrSQE-KD lines, CrSQE-KD-2 and CrSQE-KD-4, as representative lines (B). Primer sequences, each gene ID in Phytozome and their encoded enzyme names are listed in S1 Table. Expression of each gene was normalized to that of CRY1. Data in all experiments indicate mean value ± SD from three biological replicates. Asterisks above the bars indicate significant differences (*p < 0.05, **p < 0.01). (TIF) [file pone.0120446.s006.tif]
