# Supplementary material for: Accumulation of Squalene in a Microalga Chlamydomonas reinhardtii by Genetic Modification of Squalene Synthase and Squalene Epoxidase Genes
Source: PLoS One. 2015 Mar 12;10(3):e0120446. doi: 10.1371/journal.pone.0120446 (PMC4357444; doi:10.1371/journal.pone.0120446)
Supplement: Supplementary file 2 [file pone.0120446.s007.doc]

S1 Table. Primer sequences for qRT-PCR.

Gene name	Gene ID in Phytozome	Enzyme name	Forward (5′-3′)	Reverse (5′-3′)
CrDXS	Cre07.g356350	1-Deoxy-D-xylulose 5-phosphate synthase	AACAATGAGCAGCTGAAGCA	TAGTGCATAGCCACCGTCAG
CrDXR	Cre12.g546050	1-Deoxy-D-xylulose 5-phosphate reductoisomerase	ATGATCGAGACGCAGGACTC	GAGGTTGTTGGCCTTGGTAA
CrCMS	Cre16.g679669	2-C-methyl-D-erythriol 4-phosphate cytidyltransferase	GCTCGGCGCTAGTAGTGAAC	GCGAAGCTCCAGATACTGCT
CrCMK	Cre02.g145050	4-Diphosphocytidyl-2-C-methyl-D-erythritol 2-phosphate kinase	TTTCGCCCTCAAAGATCAAC	GGTGGTCTCGAACTCCATGT
CrMECPS	Cre12.g503550	2-C-methyl-D-erythritol 2,4-cyclodiphosphate synthase	GAGAACATCCGCAACAACCT	CAGGCTATCCACCTTCTCGT
CrHDS	Cre12.g490350	4-Hydroxy-3-methylbut-2-en-1-yl diphosphate synthase	CGAGCTGATTGAGGAGACCT	GTCGCCGTAGTAGGACAGGA
CrIDS	Cre08.g372950	Isopentenyl diphosphate synthase	CTTCAACAGCTCCAACACCA	CCAGCTTGTGCAGGATAACA
CrIDI	Cre11.g467544	Isopentenyl diphosphate isomerase	CCTCCTCCTTCTCCTTCCTC	ACGTAGTCCACCTCGTGCTC
CrGPS	Cre12.g511700	Geranyl pyrophosphate synthase	TGGAGGGCTTATTTCAGCAG	CAGCTGTGAGGAACAAACGA
CrFPS	Cre03.g207700	Farnesyl diphosphate synthase	GGCAAGATTGGTACGGACAT	GCCTTGATGACCTCCTTCTG
CrSQS	Cre03.g175250	Squalene synthase	TGGCAGCATGCTACAACAAC	GAACTGCAGGAACCAGGTGT
CrSQE	Cre17.g734644	Squalene epoxigenase	TGAGGACAGCGTGATACTGC	CTTGGCTTGTGCGTATGCTA
CRY1	Cre11.g480150	Ribosomal protein S14	GAAACTATTTCGCGCGTCAC	CAGTGATGCCCAGCTCCT
