# Supplementary material for: Accumulation of Squalene in a Microalga Chlamydomonas reinhardtii by Genetic Modification of Squalene Synthase and Squalene Epoxidase Genes
Source: PLoS One. 2015 Mar 12;10(3):e0120446. doi: 10.1371/journal.pone.0120446 (PMC4357444; doi:10.1371/journal.pone.0120446)
Supplement: Supplementary file 3 [file pone.0120446.s008.doc]

S2 Table. Primer sequences for trasngenic analysis.

Primer name	Sequences (5'-3')
**For construction of plasmids for *CrSQS* expression in *E. coli***	
CrSQS-pET-F	AAGGAGATATACAT**ATG**GGAAAGTTAGGGGAGCTACT
CrSQS-pET-R	GGTGGTGGTGCTCGAGGTCGCGGGTCAGCGCGGCGC
**For construction of plasmids for *CrSQE* and *ScERG1* expression in yeast mutant KLN1**	
CrSQE-pAUR123-F	TGGTACCCGGGTCGACTTGAGG**ATG**TGTACAACCTC
CrSQE-pAUR123-R	TAGAGCTCGAGTCGAC**CTA**GGACGAGGCCAGCACGC
ScERG1-pAUR123-F	TGGTACCCGGGTCGACAGGGTC**ATG**TCTGCTGTTAA
ScERG1-pAUR123-R	TAGAGCTCGAGTCGAC**TCA**CGCTCTGTGTAAAGTGT
**For construction of plasmids for *CrSQS*, *CrSQE* and *AtSQS1* expression in onion epidermal cells**	
sGFP-CrSQS-F	GCATGGACGAGCTGTACAAG**ATG**GGAAAGTTAGGGGAGCT
CrSQS-pBI221-R	GATCGGGGAAATTCG**TCA**CGCACGCCGCAGTACCG
sGFP-CrSQSTMD-F	GCATGGACGAGCTGTACAAGATGGAGGCCAAGGTGTCAAA
CrSQE-pBI221-F	CACGGGGGACTCTAGGA**ATG**TGTACAACCTCGAGCCT
SQE300-mCherry-R	TCCTCGCCCTTGCTCACCATCACCTTGATTGCGTCAATGC
mCherry-CrSQE301-F	GCATGGACGAGCTGTACAAGTACGGCTACGCGCTGTTCAA
CrSQE-pBI221-R	GATCGGGGAAATTCG**CTA**GGACGAGGCCAGCACGC
mCherry-AtSQS1TMD-F	GCATGGACGAGCTGTACAAGCTGAAGACAAAGGTTGACAA
AtSQS1-pBI221-R	GATCGGGGAAATTCG**TCA**GTTTGCTCTGAGATATG
sGFP-pBI221-F	CACGGGGGACTCTAGGA**ATG**GTGAGCAAGGGCGAGGA
sGFP-CrSQS-R	AGCTCCCCTAACTTTCCCATCTTGTACAGCTCGTCCATGC
sGFP-CrSQSTMD-R	TTTGACACCTTGGCCTCCATCTTGTACAGCTCGTCCATGC
SQE300-mCherry-F	GCATTGACGCAATCAAGGTG**ATG**GTGAGCAAGGGCGAGGA
mCherry-SQE301-R	TTGAACAGCGCGTAGCCGTACTTGTACAGCTCGTCCATGC
mCherry-AtSQS1TMD-R	TTGTCAACCTTTGTCTTCAGCTTGTACAGCTCGTCCATGC
**For overexpression of *CrSQS* in *C. reinhardtii* parental line UVM4**	
CrSQSox-F	CACAACAAGCCCATATGGGAAAGTTAGGGGAGCTAC
CrSQSox-R	CCTCGAGTCTAGATCTCGCACGCCGCAGTACCGCCA
PsaD-F	GCGGCGTGATTATTGGTATT
CrSQSox-02R	GCCACACGTTGCCTAAGAAT
**For knockdown of *CrSQE* in *C. reinhardtii* parental line UVM4**	
CrSQEKD-F[a]	ctagtACGCTCGCTTCATCCGGAAGAtctcgctgatcggcaccatgggggtggtggtgatcagcgctaTCTTTTGGATGAAGCGAGCGTg
CrSQEKD-R[a]	ctagcACGCTCGCTTCATCCAAAAGAtagcgctgatcaccaccacccccatggtgccgatcagcgagaTCTTCCGGATGAAGCGAGCGTa
AmiRNAprec	GGTGTTGGGTCGGTGTTTTTG
pChlamiRNA3-R	GCGGCCGCATTGCACAGTCA

[a]Uppercase letters indicate microRNA and its other strand sequences.

Translational start ATG and the complementary sequence of stop codon are shown in bold letters.
