# Supplementary material for: Accumulation of Squalene in a Microalga Chlamydomonas reinhardtii by Genetic Modification of Squalene Synthase and Squalene Epoxidase Genes
Source: PLoS One. 2015 Mar 12;10(3):e0120446. doi: 10.1371/journal.pone.0120446 (PMC4357444; doi:10.1371/journal.pone.0120446)
Supplement: Supplementary file 4 [file pone.0120446.s009.doc]

S3 Table. Accession numbers of SQS and SQE sequences used for alignment in S1 Fig.

Gene name	Species	Accession number[a]
BbSQS	*Botryococcus braunii*	Q9SDW9.1 GI:75265712
AtSQS1	*Arabidopsis thaliana*	NP_195190.1 GI:15236168
HsSQS	*Homo sapiens*	NP_004453.3 GI:67089147
ScERG9	*Saccharomyces cerevisiae*	AAA34597.1 GI:171481
AtSQE1	*Arabidopsis thaliana*	NP_564734; GI:18406296
HsSQE	*Homo sapiens*	NP_003120.2 GI:62865635
RnSQE	*Rattus norvegicus*	NP_058832.1 GI:8394354
ScERG1	*Saccharomyces cerevisiae*	NP_011691.1 GI:6321614

[a]Accession numbers in Genbank/EMBL/DDBJ database are shown for each gene sequence.
